# The anti-psychotic drug pimozide is a novel chemotherapeutic for breast cancer

**DOI:** 10.18632/oncotarget.26175

**Published:** 2018-10-09

**Authors:** EL-Habib Dakir, Adam Pickard, Kirtiman Srivastava, Cian M. McCrudden, Stephane R. Gross, Stephen Lloyd, Shu-Dong Zhang, Andriana Margariti, Richard Morgan, Philip S. Rudland, Mohamed El-Tanani

**Affiliations:** ^1^ Center for Cancer Research and Cell Biology, Queen’s University, Belfast, UK; ^2^ Instituto de Biología Molecular y Celular del Cáncer, Centro de Investigación del Cáncer, Consejo Superior de Investigaciones Científicas (CSIC), Universidad de Salamanca, Salamanca, Spain; ^3^ School of Pharmacy, Queen’s University Belfast, Belfast, UK; ^4^ School of Life and Health Sciences, Aston University, Birmingham, UK; ^5^ School of Medicine, Animal Facility, Queen’s University Belfast, Belfast, UK; ^6^ Northern Ireland Centre for Stratified Medicine, Biomedical Sciences, University of Ulster, UK; ^7^ Center of Experimental Medicine, Queen’s University Belfast, Belfast, UK; ^8^ Institute of Cancer Therapeutics, University of Bradford, Bradford, UK; ^9^ Institute of integrative Biology, University of Liverpool, Liverpool, UK

**Keywords:** pimozide, breast cancer, DSB, apoptosis, xenograft

## Abstract

Pimozide, an antipsychotic drug of the diphenylbutylpiperidine class, has been shown to suppress cell growth of breast cancer cells *in vitro*. In this study we further explore the inhibitory effects of this molecule in cancer cells. We found that Pimozide inhibited cell proliferation in a dose- and time-dependent manner in MDA-MB-231 breast cancer cells and A549 lung cancer cells. Furthermore, we found that Pimozide also promoted apoptosis as demonstrated by cell cycle arrest and induction of double-strand DNA breaks but did not result in any effect in the non-transformed MCF10A breast cell line. In order to shed new lights into the molecular pathways affected by Pimozide, we show that Pimozide downregulated RAN GTPase and AKT at both protein and mRNA levels and inhibited the AKT signaling pathway in MDA-MB-231 breast cancer cells. Pimozide also inhibited the epithelial mesenchymal transition and cell migration and downregulated the expression of MMPs. Administration of Pimozide showed a potent *in vivo* antitumor activity in MDA-MB-231 xenograft animal model and reduced the number of lung metastases by blocking vascular endothelial growth factor receptor 2. Furthermore, Pimozide inhibited myofibroblast formation as evaluated by the reduction in α-smooth muscle actin containing cells. Thus, Pimozide might inhibit tumor development by suppressing angiogenesis and by paracrine stimulation provided by host reactive stromal cells. These results demonstrate a novel *in vitro* and *in vivo* antitumor activity of Pimozide against breast and lung cancer cells and provide the proof of concept for a putative Pimozide as a novel approach for cancer therapy.

## INTRODUCTION

Antipsychotic drugs (APDs) are the first pharmacotherapeutic line of treatment for schizophrenia. They are also widely used for the treatment of bipolar disorder and other idiopathic psychotic disorders. The primary molecular targets of ADPs are the neurotransmitter receptors, dopamine receptors and their interaction are thought to be important for the “normalization” of neurotransmitter imbalances that are associated with specific symptoms and the acute manifestation of schizophrenia. Pimozide is a neuroleptic drug selectively blocks dopamine receptor D2 (DRD2) [1, 2], used to treat a number of mental/mood disorders (chronic schizophrenia), as well as other psychotic disorders, as it reduces dopamine activity and consequently decreases excitation, agitation, hypermobility, and abnormal conditions associated with excess energy [3, 4]. It has long been suggested that patients with schizophrenia have a reduced incidence of cancer compared to the general population [5, 6]. Many epidemiological studies have been conducted to investigate this possible link, but cancer risk in schizophrenic patients remains a controversial issue [7–9], although antipsychotic drugs have been suggested as possible mediators of this effect [5, 10, 11].

Ran GTPase (Ran), which belongs to the Ras superfamily of small GTPases, and the proteins which regulate GTP binding and hydrolysis have a well-defined role in nuclear transport [12, 13]. Ran itself is an abundant GTPase that is highly conserved in eukaryotic cells and has been implicated in many aspects of nuclear structure and function, cell cycle regulation, nuclear transport, and cell transformation [14, 15].

In this work, we identified Pimozide as a potential inhibitor of Ran using connectivity map analysis [16] of the breast cancer cell line MDA-MB-231, previously silenced by shRNA against the expression of the Ran gene. Pimozide was previously shown to inhibit proliferation of the human breast cancer-derived cell line MCF-7 *in vitro* by blocking estradiol-induced growth [17]. Pimozide has gained attention as an anti-cancer agent by acting as a STAT5 inhibitor in chronic myelogenous leukemia cells [18], as well as an inhibitor of STAT3 signaling pathway in hepatocellular carcinoma and suppressing cancer stem-like cell maintenance [19]. Previous work has demonstrated that Pimozide inhibited cell growth of Hepatocellular carcinoma (HCC) cells by disrupting the Wnt/β-catenin signaling pathway and reducing epithelial cell adhesion molecule (EpCAM) expression [20]. To explore the mechanisms involved in Pimozide inhibition of cancer and metastasis, we have analyzed the effect of Pimozide on breast cancer cell lines *in vitro* and breast cancer xenograft models *in vivo*. In this study, we demonstrate that Pimozide decreases *Ran* mRNA expression and reduces the expression of AKT and phosphorylation of VEGFR2 in breast cancer cell lines and in Human Umbilical Vein Endothelial Cells (HUVECs), leading to increased caspase-3 activation and apoptotic cell death. Pimozide also causes a reduction in cell proliferation, cell migration and invasion *in vitro* and of lung metastasis *in vivo*. Taken together, these results suggest that Pimozide may be of practical value in the management of human breast cancer, and could play a pivotal role in inhibiting cellular proliferation, migration, and metastasis.

## RESULTS

### Connectivity map (Map) analysis

The Java application sscMap, which was bundled with over 6000 reference gene expression profiles for over 1000 compounds as its core database, was queried to identify compounds that had significant connections to the expression of Ran in the human invasive breast cancer cell line MDA-MB-231, previously silenced with shRNA against the *Ran* gene. These 1000 distinct small molecule perturbagens, selected to represent a broad range of activities, include U.S. Food and Drug Administration (FDA)–approved drugs and nondrug bioactive ‘‘tool’’ compounds. The top candidate compounds that had significant connections to Ran expression are listed in Table 1. Highlighted in blue are drugs that are predicted to have inhibitory effects on the expression of Ran, whilst those in red are predicted to have an enhancing effect on Ran overexpression. As can be seen, Pimozide was highly ranked (P = 0.00001, z-score = -4.8028) compared to other drugs (Table 1).

**Table 1:** Connectivity map analysis of human breast cancer MDA-MB-231 cells after Ran silencing using shRNA

Compound	Replicates	Set score	p-value	stdev	zscore
**Pimozide**	4	-0.8084	0.00001	0.16833	-4.8028
**SC-58125**	4	-0.7283	0.00001	0.16583	-4.392
**Pimethixene**	3	-0.8298	0.00004	0.1952	-4.2507
**Fluorocurarine**	4	-0.6746	0.00004	0.16231	-4.1563
**Chlorphenesin**	4	-0.8019	0.00001	0.19792	-4.0514
**Ouabain**	4	-0.7897	0.0001	0.19517	-4.0463
**Emetine**	4	-0.8836	0.00002	0.22767	-3.8812
**Vinpocetine**	4	-0.8086	0.00002	0.2174	-3.7193
**HNMPA-(AM)3**	1	-0.9356	0.00002	0.28851	-3.2427
**Oxamic acid**	1	-0.9337	0.00002	0.28851	-3.2363
**Tomelukast**	1	0.93609	8.00E-05	0.28851	3.24454
**Equilin**	5	0.73566	2.00E-05	0.17639	4.17066
**Promethazine**	4	-0.7215	0.00004	0.18346	-3.9326
**Etacrynic acid**	3	-0.7908	0.00002	0.20202	-3.9146
**Cortisone**	3	-0.8615	0.00002	0.2284	-3.7716
**Oxymetazoline**	4	-0.8091	0.00001	0.2239	-3.6139
**Sulfamethoxypyridazine**	5	-0.8193	0.00002	0.2271	-3.6074
**Dipyridamole**	6	-0.734	0.00006	0.20596	-3.5638
**Cotinine**	6	-0.7913	0.00006	0.22281	-3.5512
**Ronidazole**	3	-0.8361	0.00008	0.25165	-3.3224
**Lobeline**	4	0.43885	8.00E-05	0.12488	3.51418
**Docosahexaenoic acid ethyl ester**	2	0.82579	0.00001	0.22997	3.59087
**Alpha-yohimbine**	3	0.75844	0.00012	0.20143	3.76532
**Esculetin**	3	0.81007	6.00E-05	0.21378	3.78931

### Pimozide inhibits cancer cell growth *in vitro* and results in DNA damage

**Figure 1:**
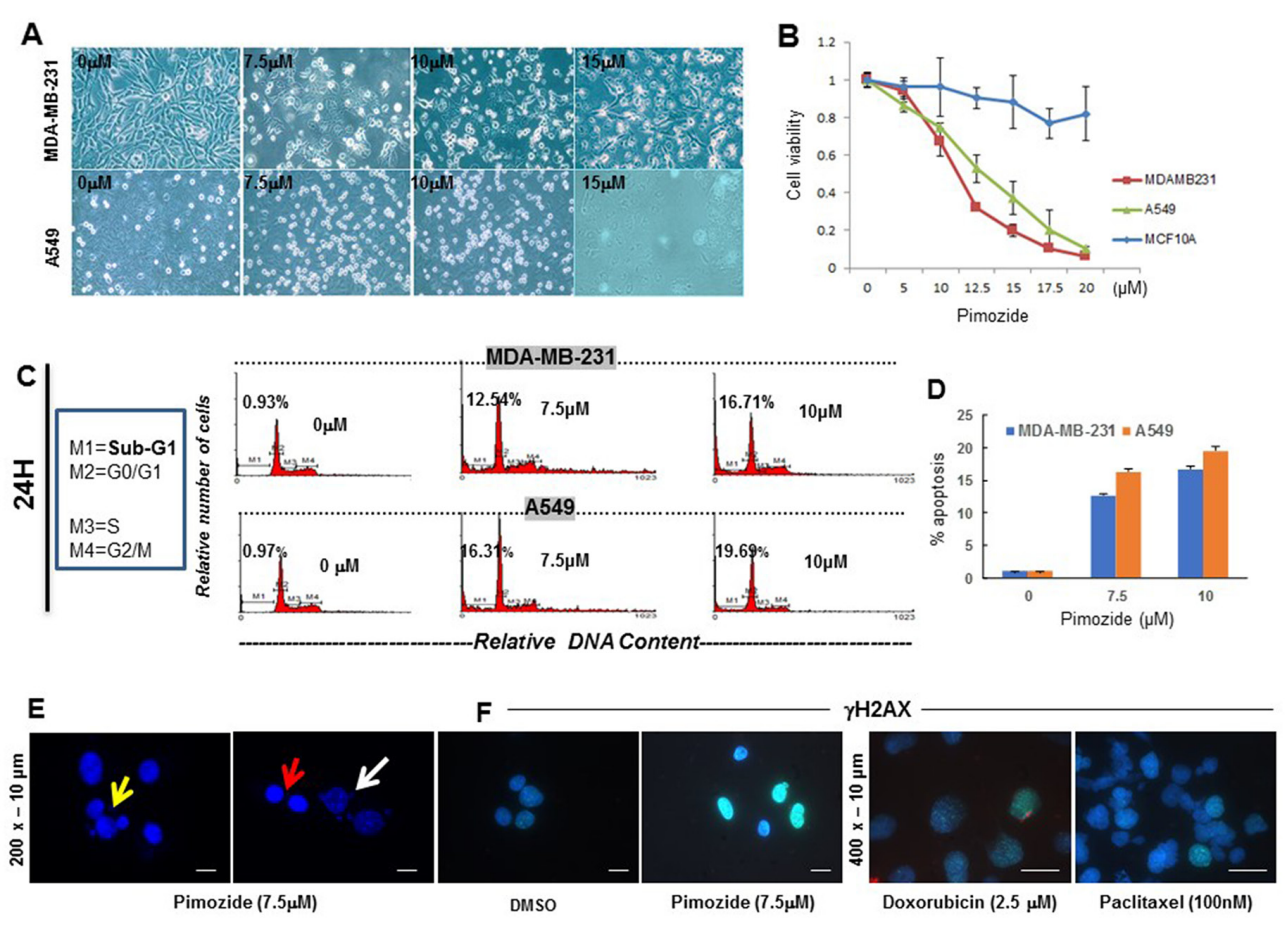

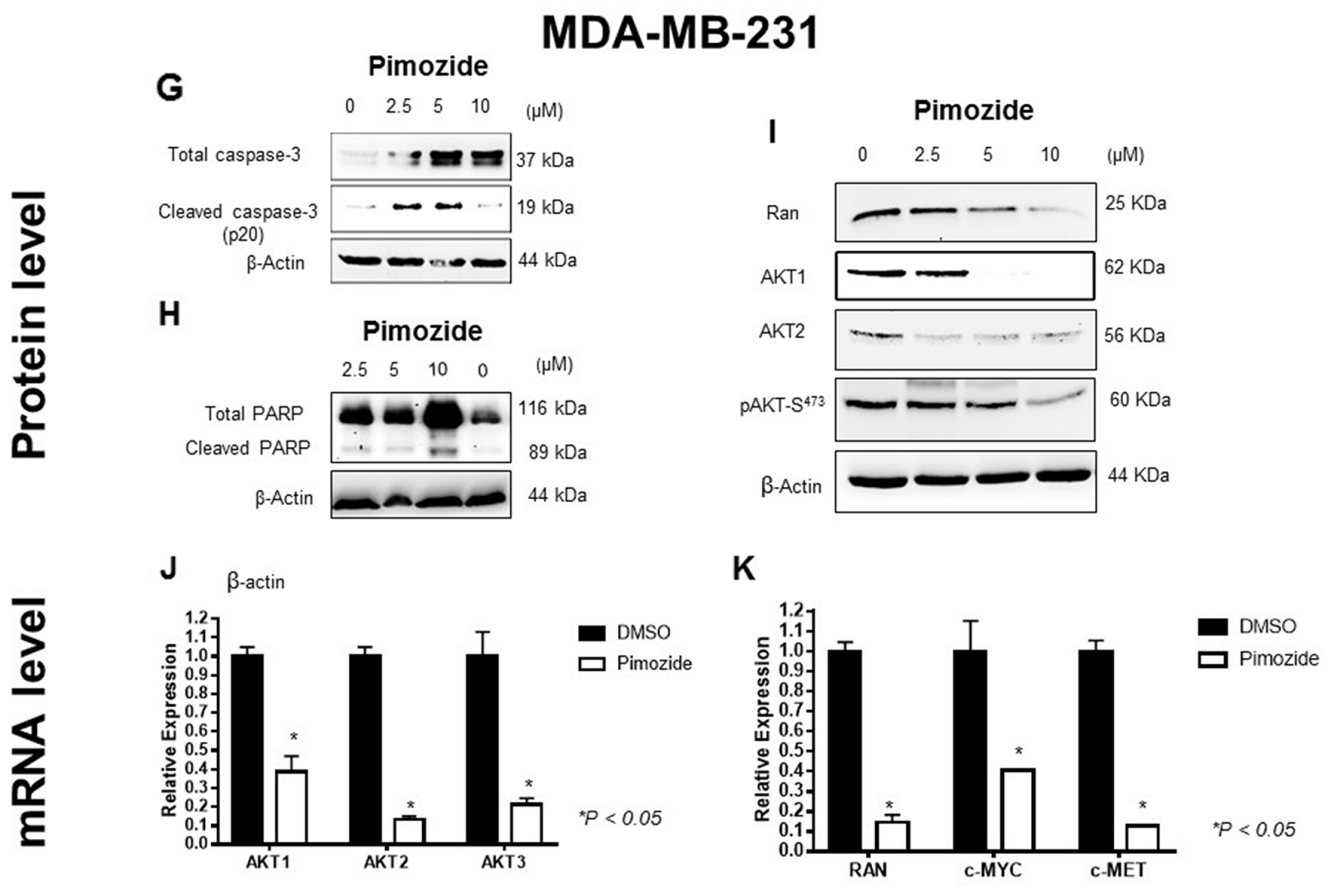
Pimozide inhibits cell proliferation in a dose- and time-dependent manner by inducing cell cycle arrest and DNA double strand breaks (DSBs) **(A)** Phase contrast micrograph showing cell morphology of human breast cancer MDA-MB-231 and lung cancer A549 cells treated with Pimozide at different doses (μM) for 24 hours. Scale bar 100 μm. **(B)** Viability of MDA-MB-231 cancer cells, MCF10A normal breast cells, and A549 lung cancer cells after 48 hours treatment with Pimozide. **(C)** Cell cycle profiles. DNA content of fixed, propidium iodide-stained cells was analyzed by flow cytometry. MDA-MB-231 and A549 cells were incubated in the absence (control 0 μM of Pimozide with DMSO) or in the presence of Pimozide at different doses for 24 hours, and the percentage of cells in sub-G1 (M1) (hypodiploidy), G0/G1 (M2), S (M3), and G2/M (M4) phases calculated using flow cytometry. The data gatings are indicated for each DNA histogram for M1, M2, M3 and M4. The percentage of cells with a DNA content less than G1 (sub-G1) is indicated in each histogram. The cell cycle profiles shown, with the sub-G1 population indicated, are representative of three experiments performed. **(D)** Cell cycle profile (Sub-G1) summarized using a histogram. MDA-MB-231 and A549 cells were treated with different doses of Pimozide for 24 hours, and the percentage of cells in the sub-G1 phase of the cell cycle (dead and dying cells) was quantitated by flow cytometry. Data shown are means ± SD of three independent experiments, ^**^, *P < 0.01*, Student’s *t*-test. **(E & F)** DNA damage response (DDR) measured after treatment with Pimozide, Doxorubicin (Doxo) and Paclitaxel (Pac). (E) Representative fields of DAPI-stained DNA showing nuclear damage after 7.5 μM Pimozide treatment. Some nuclei show signs of dynamic DNA membrane blebbing (white arrow) and fragmentation after treatment for 48 hours, whilst other nuclei remained unchanged. There is also fragmented DNA outside (red arrow), and inside the nucleus (yellow arrow) suggesting chromatin condensation after treatment. Magnification 200x. Scale bar 10 μm. (F) Staining for histone γH2AX in MDA-MB-231 cells treated with 7.5 μM Pimozide or with 2.5 μM Doxorubicin or 100 nM Paclitaxel for 24 hours, images are representative of three experiments performed. Magnification 200x for DMSO and Pimozide treatment. Scale bar 10 μm. Magnification 400x. Scale bar 10 μm for Doxorubicin and Paclitaxel treatment. Pimozide reduced the protein and RNA expression of *AKT* and *Ran*, as well as RNA expression of *cMYC* and *cMET*. **(G)** Western blotting analysis of Caspas-3 in MDA-MB-231 treated with Pimozide at different doses for 24 hours. β-Actin was used as a loading control. Data shown are representative of three experiments performed. **(H)** Western blotting analysis of PARP in MDA-MB-231 treated with Pimozide at different doses for 24 hours. β-Actin was used as a loading control. Data shown are representative of three experiments performed. **(I)** Western blotting analysis of Ran, AKT1, AKT2 and pAKT in MDA-MB-231 treated with Pimozide at different doses for 24 hours. β-Actin was used as a loading control. Data shown are representative of three experiments performed. **(J)** Relative mRNA expression of all *AKT* isoforms in MDA-MB-231 cells either untreated or treated with Pimozide 7.5 μM for 24 hours. Data shown are representative of three experiments performed. **(K)** Relative mRNA expression of *Ran*, *c-MYC* and *c-MET* in MDA-MB-231 cells treated with 7.5 μM Pimozide for 24 hours. Data shown are representative of three experiments performed.

To investigate whether Pimozide exerts direct anti-proliferative and pro-apoptotic effects, and causes DNA damage, we treated human invasive breast cancer MDA-MB-231, normal breast MCF10A, and lung adenocarcinoma A549 cells with this drug at different doses for 24 or 48 hours, and cell morphology was observed after 24 hours (Figure 1A). Cell viability was assessed after treatment with different doses of Pimozide after 48 hours (Figure 1B). Whilst the survival of both cancer cell lines was significantly affected by Pimozide, MCF10A was relatively insensitive and showed little cell death (∼5% cell death) even with 20 μM Pimozide (which caused >90% cell death in MDA-MB-231 and A549 cells). We next characterized the apoptotic cell death induced by Pimozide in MDA-MB-231 and A549 cells by using several *bona fide* markers of apoptosis. Cell cycle analyses by flow cytometry showed that Pimozide treatment for 24 hours rendered an increase in the sub-G1 cell population, representing apoptotic cells (Figure 1C, 1D), and described in Supplementary Table 1, available online. This apoptotic response, detected by the appearance of a sub-G1 population in cell cycle analysis, which is indicative of DNA degradation and DNA damage response (DDR) in MDA-MB-231 cells, was further supported by the internucleosomal DNA fragmentations (red arrow) and chromatin condensation (white arrow), and DNA blebbing (yellow arrow) detected after 48 h incubation with 7.5 μM Pimozide (Figure 1E). There was also evidence of double-strand DNA breaks (DSBs) measured by an increase of phosphorylated H2A histone family member X (γ-H2AX) expression after Pimozide treatment, to a greater extent than that observed with Doxorubicin and Paclitaxel (Figure 1F). The normal breast cell line MCF10A showed no evidence of DDR at this dose or even at 15 μM of Pimozide (data not shown). In addition, we found that Pimozide induced caspase-3 activation, as assessed by cleavage of procaspase-3 into their respective p20 active forms (Figure 1G), as well as by proteolysis of the caspase-3 substrate 116 kDa-poly(ADP-ribose) polymerase (PARP) into the 86-kDa cleaved form of PARP in MDA-MB-231 cells as assessed by Western blot (Figure 1H).

### Pimozide reduced expression of *Ran* and *AKT* isoforms at both the mRNA and protein level and affected *cMET* and *cMYC* expression

Western blotting for Ran, AKT1 and AKT2 isoforms revealed reduced protein expression after Pimozide treatment; Ran expression was reduced by 45% after treatment with 5 μM Pimozide and by 87% after treatment with 10 μM Pimozide. AKT1 expression was almost completely abolished after treatment with 5 μM Pimozide and AKT2 expression was reduced by 72% after treatment with 2.5 μM Pimozide. There was also a significant decrease (78%) in AKT phosphorylation after treatment for 24 hours with increasing concentrations of Pimozide up to 10 μM. (Student’s *t* test *P < 0.05*) (Figure 1I). In addition, the RNA expression of *AKT* isoforms (*AKT1*, *AKT2* and *AKT3*) was measured (Figure 1J), revealing a significant reduction in the RNA expression of *AKT1* (1.5 fold), *AKT2* (9 fold), and *AKT3* (4.76 fold) (Student’s *t* test *P < 0.*05) after treatment of MDA-MB-231 cells with 7.5 μM Pimozide. There was also a significant reduction in the RNA expression of *Ran* (6.7 fold), *c-Myc* (2.5 fold), and *c-Met* (9 fold) (Student’s *t* test *P < 0.05*) after 24 hours of Pimozide treatment of MDA-MB-231 cells with the same dose of Pimozide (7.5 μM) (Figure 1K).

### Pimozide inhibits the growth of invasive human breast cancer MDA-MB-231 xenografts in SCID mice

**Figure 2:**
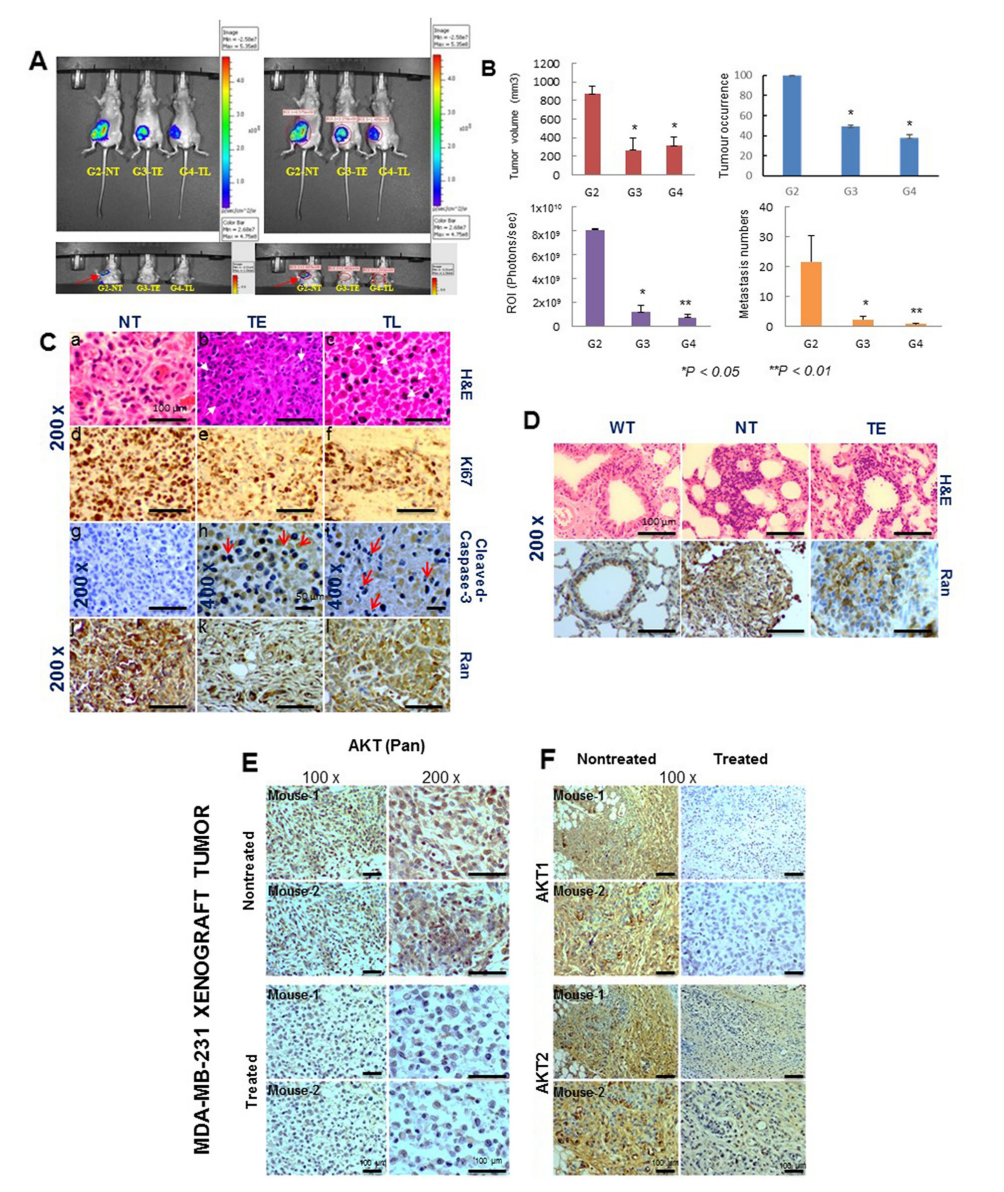
Pimozide reduces tumor burden, cell proliferation, and the number of lung metastases in a nude mice xenograft model system **(A)** *In vivo* bioluminescence imaging system (IVIS) of MDA-MB-231-Luc (D3H2LN) xenografts in SCID mice. Ventral (upper left panels) images taken over time from representative mice (10 imaged). Upper right panels show the Region of Interest (ROI) in localized tumors from representative mice (10 imaged). Pseudo color scale bars were consistent for all imaged ventral views in order to show relative changes at metastatic sites over time, lower left panel images show metastatic foci in control mice (G2NT), and the lower right panel shows the ROI in different mice (red arrow). **(B)** Characterization of tumors in untreated (PBS) and treated (Pimozide) groups by plotting tumor volume (mm^3^), tumor occurrence (%), ROI (photons/sec) and lung metastatic foci numbers with significance values at autopsy. Data shown are means ± SD (Student’s *t* test ^*^P <0.05, ^**^P < 0.01). **(C)** Hematoxylin & eosin (H&E) staining of xenograft tumors in untreated (NT) (a), earlier treated (TE) (b) and late treated (TL) (c) mice. Magnification 200x. Scale bar 100 μm. Immunohistochemical staining for Ki67 (d,e,f). Magnification 200x. Scale bar 100 μm, cleaved caspase-3 (g,h,i). Magnification 200x. Scale bar 100 μm (g). Magnification 400x. Scale bar 50 μm (h, i) and Ran (j,k,l) in NT, TE and TL mice. Magnification 200x.Scale bar 100 μm **(D)** H&E staining of representative lung metastases from WT, NT, and TE mice. Ran immunostaining was assessed in wild type lung and lung metastases (NT and TE), magnification 200x. Scale bar 100 μm. **(E & F)** Immunohistochemical staining for AKT in MDA-MB-231 xenograft tumors. (E) Immunohistochemical staining for AKT (Pan). Scale bar 100 μm. (F) AKT-isoforms (AKT1, AKT2) in untreated and treated mice breast xenografts tumors showing a representative result for 10 sections per group. Magnification 100x. Scale bar 100 μm.

We tested the antitumor effects of Pimozide on the growth of human invasive breast cancer cell line MDA-MB-231 xenografted tumors *in vivo*. Breast cancer cells were injected orthotopically into the mammary fat pat of SCID mice as described in Supplementary Figure 1A, available online. These cells were allowed to develop into mammary tumor xenografts, mice were divided into 4 groups, Group-1 as nontreated control without tumors, Group-2 with nontreated tumors (NT), Group-3 with tumors treated the day after implantation with Pimozide 20 mg/kg (TE) and Group-4 with tumors that were treated with the same dose when the tumor was palpable (TL). The volume of tumors in the control group increased significantly between day 0 and day 14 (mean volume = 100 mm^3^), a representative mouse xenograft of each group (2, 3 and 4), is shown in Figure 2A. G3TE and G4TL mice are shown in Supplementary Figure 1B, available online. Beginning on day 14, mice in Group-4 were treated by i.p. injection of 20 mg/kg Pimozide for 5 days a week. Tumor volumes were reduced by 65% (*P < 0.05*) (Group-3) and by 60% (*P < 0.05*) (Group-4) (Figure 2B), although tumor occurrence in the treated groups were reduced by 55% (*P < 0.05*) (Group-3) and 61% (*P < 0.05*) (Group-4) (Figure 2B).

Tumor metastases were found only in the lung after a complete necropsy, and there were 94% (*P < 0.05*) (Group-3) and 92% (*P < 0.01*) (Group-4) fewer metastases compared to the nontreated mice. The region of interests (ROI) finding for the same mice revealed a drastic reduction in light output between treated (7 and 7.5 fold, Student’s *t* test *P < 0.05*) (Groups-3, 4, respectively), and untreated mice (Group-2) (Figure 2B, left lower panel). Untreated tumor-bearing mice and treated mice (NT, TE and TL) were autopsied and the tumor sections were stained with Hematoxylin and Eosin. This showed the presence of nuclear fragmentations (red arrow) in tumors from TE mice and irregular pyknotic nuclei (white arrow) in tumors from TL mice, characteristic of cell death by apoptosis (Figure 2C–2b, c). Magnification 200x. Scale bar 100 μm. A strong cell proliferation activity, as assessed by Ki67 staining, was determined in the tumors from untreated mice (Figure 2C–2d), while cell proliferation was low in tumors from TE and TL mice (Figure 2C–2e,f)) as shown by a decreased stained cells for Ki67. There was also increased staining for cleaved-caspase-3 in tumors from both early- and late treated mice compared to tumors from untreated mice (Figure 2C–2g,h,i), together with an increase in apoptosis index *in vivo* as indicated by the red arrows. These results indicate that Pimozide treatment induces apoptosis in the MDA-MB-231 tumors *in vivo*. Staining for Ran protein was reduced in tumors in the TE and TL treated groups (Figure 2C–2j,k,l). Lung metastases were assessed at the end of experiment, and the highest number of lesions were found in the untreated mice (Figure 2D), with fewer lesions in early and late treated mice (Supplementary Figure 2A, 2B, available online) with very few focal metastases in the airway epithelium of TE mice (Figure 2D). Carcinoma cells stained for Ran was checked using immunohistochemistry in metastatic lung foci, revealing a higher staining in lung metastases from nontreated mice (NT) compared to those from treated mice (TE) (Figure 2D).

### Pimozide reduced expression of AKT1 and AKT2 in tumors of breast cancer xenograft tumors

To study the biological mechanisms underlying the growth inhibitory effects of Pimozide, the AKT signaling pathway, which plays a role during tumor development and angiogenesis, was assessed by examining the expression of two AKT isoforms (AKT1, AKT2) using immunohistochemistry. AKT (Pan) was absent from treated tumors but present in untreated tumors (Figure 2E), AKT1 expression was completely suppressed in treated tumors (TE and TL) compared to untreated tumors, whilst slight detection of AKT2 was observed in treated tumors, probably due to artefacts and staining techniques (Figure 2F).

### Effect of pimozide on cell migration, invasion and metalloproteinase (MMP) expression

**Figure 3:**
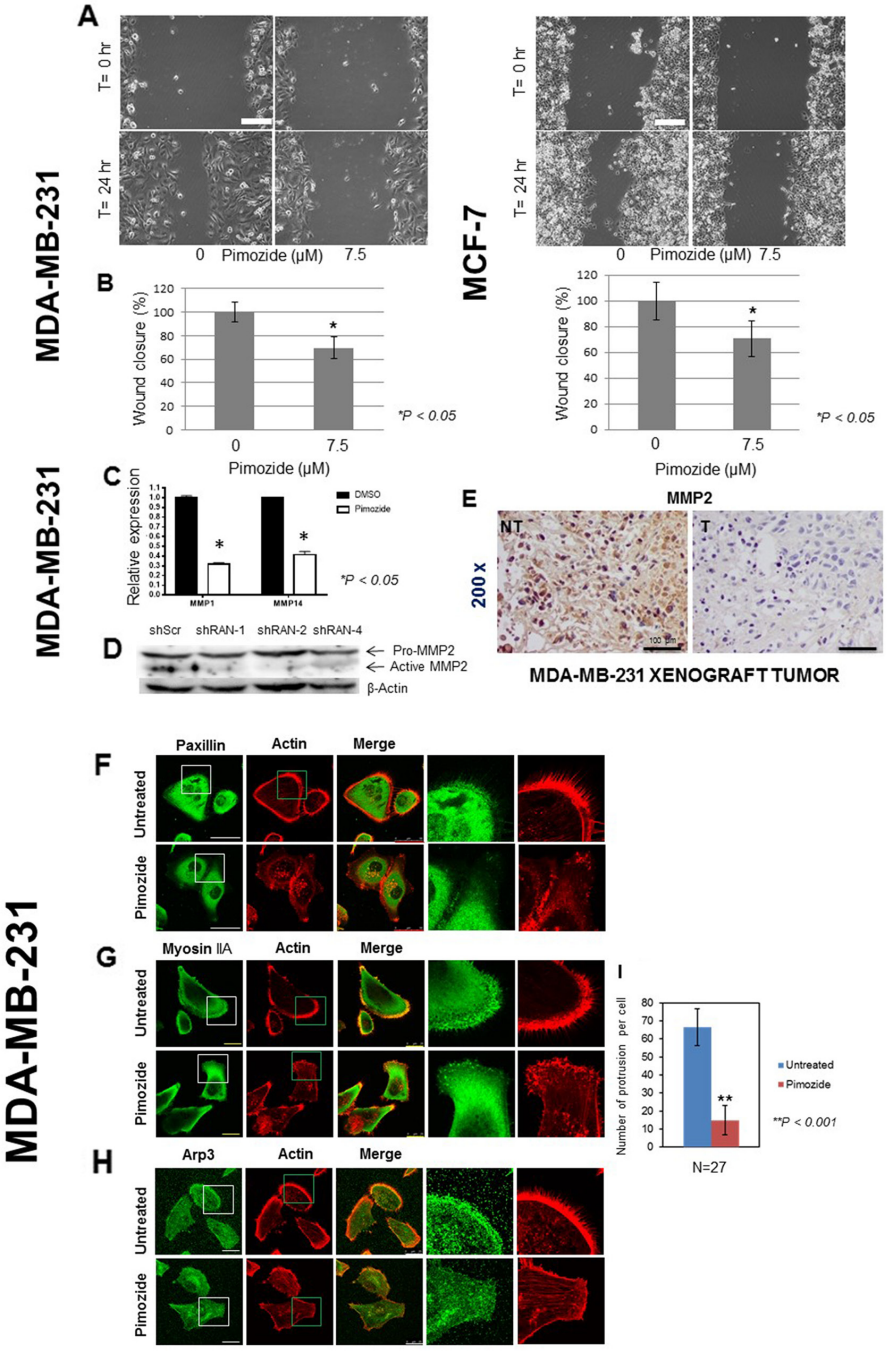
Pimozide suppresses the migration of breast cancer cells and reduces the expression of metalloproteinases (MMPs) *in vitro* and *in vivo* **(A)** MDA-MB-231 and MCF7 cells were grown for 48 hours. Following wounding of the cell monolayers, cells were treated with 7.5 μM Pimozide, or control (DMSO) and their migration to close the wound measured over time. Time lapse images of MDA-MB-231 cells and MCF7 cells, immediately after wounding and after 24 hours. Scale bar 100 μm. **(B)** Quantification of wound closure presented as percentage of untreated control (DMSO) set at 100%, and treated cells representing the proportion of closed wounded area at 24 hours post wounding of MDA-MB-231 and MCF7 cultures. Data shown are means ± SE of three independent experiments, ^*^, *P < 0.05*, Student’s *t*-test. **(C)** Relative mRNA expression of metalloproteinases *MMP1* and *MMP14* in MDA-MB-231 cells either untreated or treated with 7.5 μM Pimozide for 24 hours. Data shown are representative of three experiments performed. **(D)** Western blot showing protein levels of activated metalloproteinase MMP2 in cells treated with shScr, RAN-1, RAN-2, and RAN-4 shRNA for 24 hours. β-Actin was used as a loading control. Data shown are representative of three experiments performed. **(E)** Immunohistochemical staining for MMP2 in mouse tumor xenografts (NT & T). Magnification 200x. Scale bar 100 μm. Pimozide inhibits the migration of cells. Breast cancer MDA-MB-231 cells treated with 7.5 μM Pimozide or control (DMSO) were grown for 48 hours on fibronectin-coated coverslips prior to washing, fixing and immunostaining for either: **(F)** paxillin. Scale bar 50 μm, **(G)** myosin IIA, or **(H)** Arp 3. Scale bar 25 μm, together with staining for actin using rhodamine-phalloidin. Scale bar 100 μm. Data shown are means ± SD of three independent experiments, ^**^, *P < 0.001*, Student’s *t*-test. **(I)** Numbers of cellular protrusions summarized using a histogram, data shown are means ± SD of three independent experiments (n = 27), ^**^, *P < 0.001*, Student’s *t*-test.

The consequence of 7.5 μM Pimozide treatment on the migration [21–23] of MDA-MB-231 and MCF7 breast cancer cell lines was investigated. Whilst the untreated cells had undergone collective migration into the empty space 24 hours post wounding, treated cells demonstrated a reduction in their abilities to migrate (Figure 3A) by 30% (Student’s *t* test *P < 0.05*) in both MDA-MD-231 and MCF7 breast cancer cells in the presence of Pimozide (Figure 3B).

The relative expression of *MMP1* and *MMP14* RNA was also decreased in MDA-MB-231 cells treated with 7.5 μM Pimozide, by 3.3 and 2.5 fold, respectively, compared to untreated cells after 24 hours (Figure 3C), suggesting that Pimozide exerts a negative effect on MMP levels. We tested whether downregulation of Ran expression by three shRNAs against the *Ran* gene had a direct effect on metalloproteinase levels (Figure 3D). We selected three shRNAs against *Ran* (shRAN-1, shRAN-2, and shRAN-4) that efficiency reduced Ran protein expression by western blotting. Ran silencing reduced the expression of the active form of MMP2 by 70% (ShRNA1), 80% (shRNA2), and 56% (shRNA4) (Student’s *t* test *P < 0.05*). This was confirmed *in vivo* as the immunohistochemical analysis of tumors revealed a complete loss of MMP2 after Pimozide treatment (Figure 3E).

### Pimozide inhibits migration related protein in MDA-MB-231 and A549 cancer cells

To provide further evidence of the migratory deficiencies after Pimozide treatment and possibly to provide clues about the molecular mechanisms involved, MDA-MB-231 and A549 cells were immunohistochemically stained for different markers related to cell motility, paxillin, myosin IIA, and Arp3 (Figure 3F, 3G, 3H). Paxillin is a focal adhesion-associated adaptor protein (FA) in a complex that acts as an interface between integrins and the actin cytoskeleton [24], myosin IIA is usually found in the lamella of motile cells and is mainly responsible for the contractile forces required during the organization of actin stress fibers [25], and Arp3 is a component of the Arp2/3 complex that promotes actin nucleation and polymerization and is mainly located at the leading edge and principally the lamellipodium of migratory cells [26]. Staining MDA-MB-231 cells for all these markers demonstrated their motile and invasive nature, with clear polarization and a well-defined leading edge (Figure 3F, 3G, 3H Paxillin, Myosin IIA, and Arp staining, respectively). Lamellipodia and filopodia structures were also clearly visible along with a few actin stress fibers throughout the cell bodies (Figure 3F, 3G, 3H, Actin staining). Treatment of cancer cells with 7.5 μM Pimozide resulted in dramatic changes in the overall cytoskeletal architecture. Polarization and a defined leading edge was lost in most cancer cells, along with a significant reduction in the numbers of cellular protrusions, from an average of 65±10 (mean ± SD) per cell in untreated cells to around 15±8 (mean ± SD) in their treated counterparts (Student’s *t* test *P < 0.001*) (Figure 3I). A very high concentration of actin stress fibers and punctate actin clusters could be seen in the body of the treated cells (Figure 3F, 3G, 3H, Actin staining). There were relatively few focal adhesion complexes in the control untreated cells (which is an indication of dynamic reorganization of these protein complexes and a sign of motility), incubating the cells with Pimozide resulted in an increased number of paxillin foci at localized regions of the cell body (Figure 3F, Paxillin staining). A549 cells treated with Pimozide likewise showed dramatic changes in cell morphology with fewer lamellipodia structures and lower Arp3 expression (data not shown). Microtubule stabilization and nuclei condensation in MDA-MB-231 and A549 cells treated with either Pimozide (7.5 μM) or Paclitaxel (100 nM), were also studied (Supplementary Figure 3, available online), revealing a significant disorganization of their microtubule cytoskeleton as evidenced by extensive bundling throughout the cytoplasm and dramatic remodeling in both cell types.

### Pimozide blocks the expression of epithelial to mesenchymal transition (EMT) markers in MDA-MB-231 cancer cells

**Figure 4:**
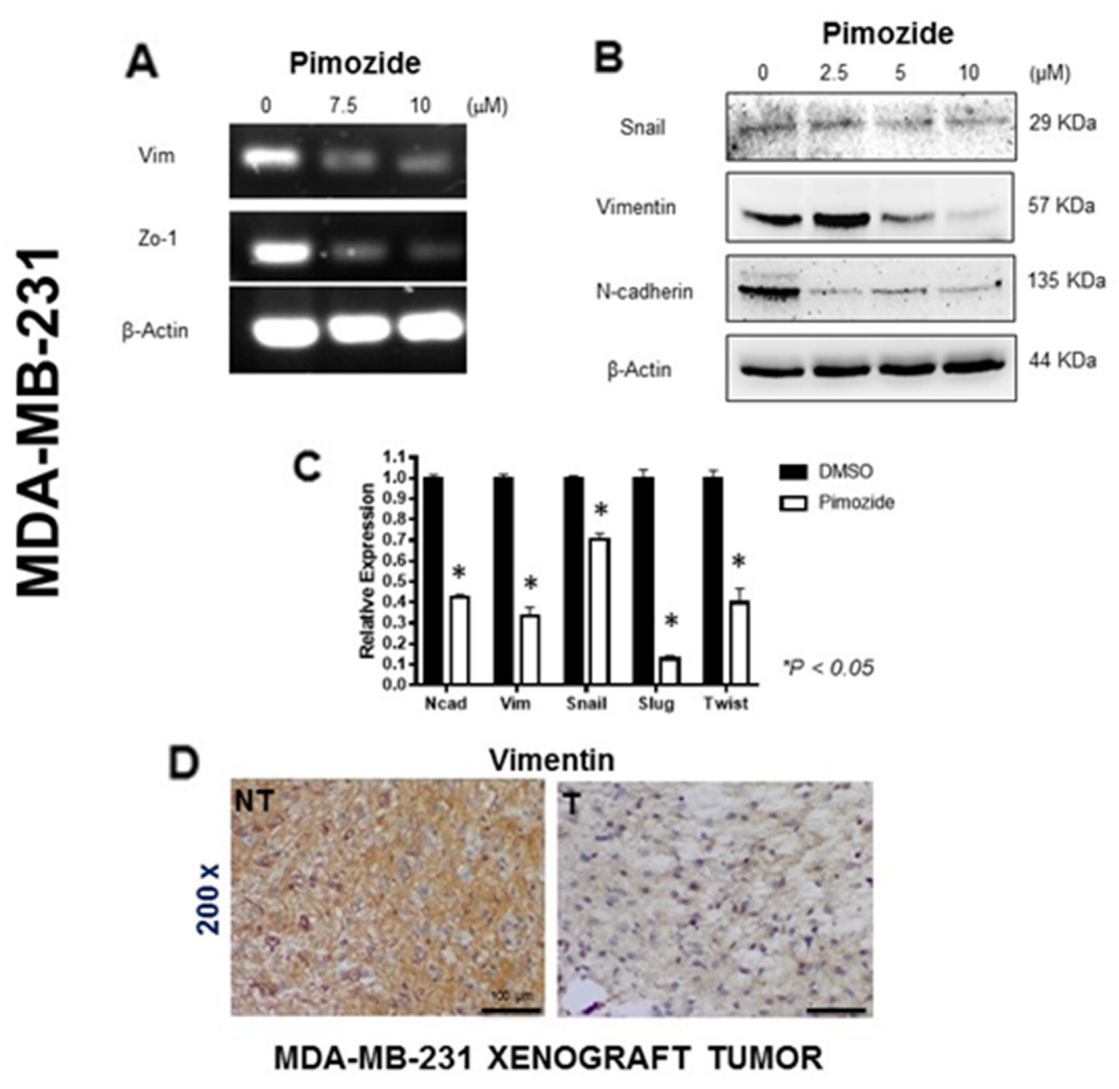
Pimozide induced epithelial mesenchymal transition (EMT) signaling pathways in MDA-MB-231 cells **(A)** RT-PCR of the EMT mRNA markers Vimentin (*Vim*) and *Zo-*1 after 24 hours treatment with Pimozide at 7.5 μM and 10 μM, showing a reduction relative to mRNA for *β-Actin*. **(B)** Western blot of Snail, Vimentin and N-cadherin proteins after 24 hours of Pimozide treatment, β-Actin was used as a loading control. Data shown are representative of three experiments performed. **(C)** Relative level of mRNA for EMT markers in MDA-MB-231 cell lines treated with 7.5 μM Pimozide for 24 hours, showing a decrease in N-cadherin (*Ncad*), Vimentin (*Vim*), *Snail*, *Slug* and *Twist* compared to untreated control (DMSO). β-Actin was used as a loading control. Data shown are representative of three experiments performed. **(D)** Immunohistochemical staining for Vimentin in tumors from untreated mice (NT) and treated mice (T) with Pimozide (a representative of 10 sections per group). Magnification 200x. Scale bar 100 μm. Data shown are representative of three experiments performed.

RT-PCR-based analysis of two EMT markers (Vimentin and Zo-1) in MDA-MB-231 breast cancer cell lines treated with Pimozide for 24 hours revealed a significant decrease in the RNA expression of both markers (Figure 4A). A western blot for Snail, Vimentin, and N-cadherin proteins also revealed that the expression of each was reduced after treatment. After treatment with 5 μM Pimozide Snail expression was reduced by 17% and Vimentin expression by 50%, whilst N-cadherin expression was reduced by 90% after treatment with only 2.5 μM Pimozide (Student’s *t* test *P < 0.05*) (Figure 4B). The relative RNA expression of EMT markers was likewise reduced after Pimozide treatment at 7.5 μM, by 2.5 fold (N-cadherin), 2.85 fold (Vimentin), 1.42 fold (Snail), 6.66 fold (Slug), and 2.5 fold (Twist) (Figure 4C), while E-cadherin expression was unchanged during the treatment (data not shown). Similar results for Vimentin were obtained in the *in vivo* study as tumors from Pimozide treated mice showed a significant decrease in staining for Vimentin (Figure 4D). The expression of additional EMT markers - FGFR2b, FGFR2c, p63 (Pan), and ACTA2 – was studied in MDA-MB-231 cells either untreated or treated with Pimozide at 7.5 μM, which revealed a significant reduction in the RNA expression of *p63* (Pan) (2 fold) and *ACTA2* (4 fold) (Supplementary Figure 4A, available online).

### Pimozide suppresses VEGF-induced cell migration and proliferation by reducing CD31 expression and downregulating AKT signaling in HUVEC cells

**Figure 5:**
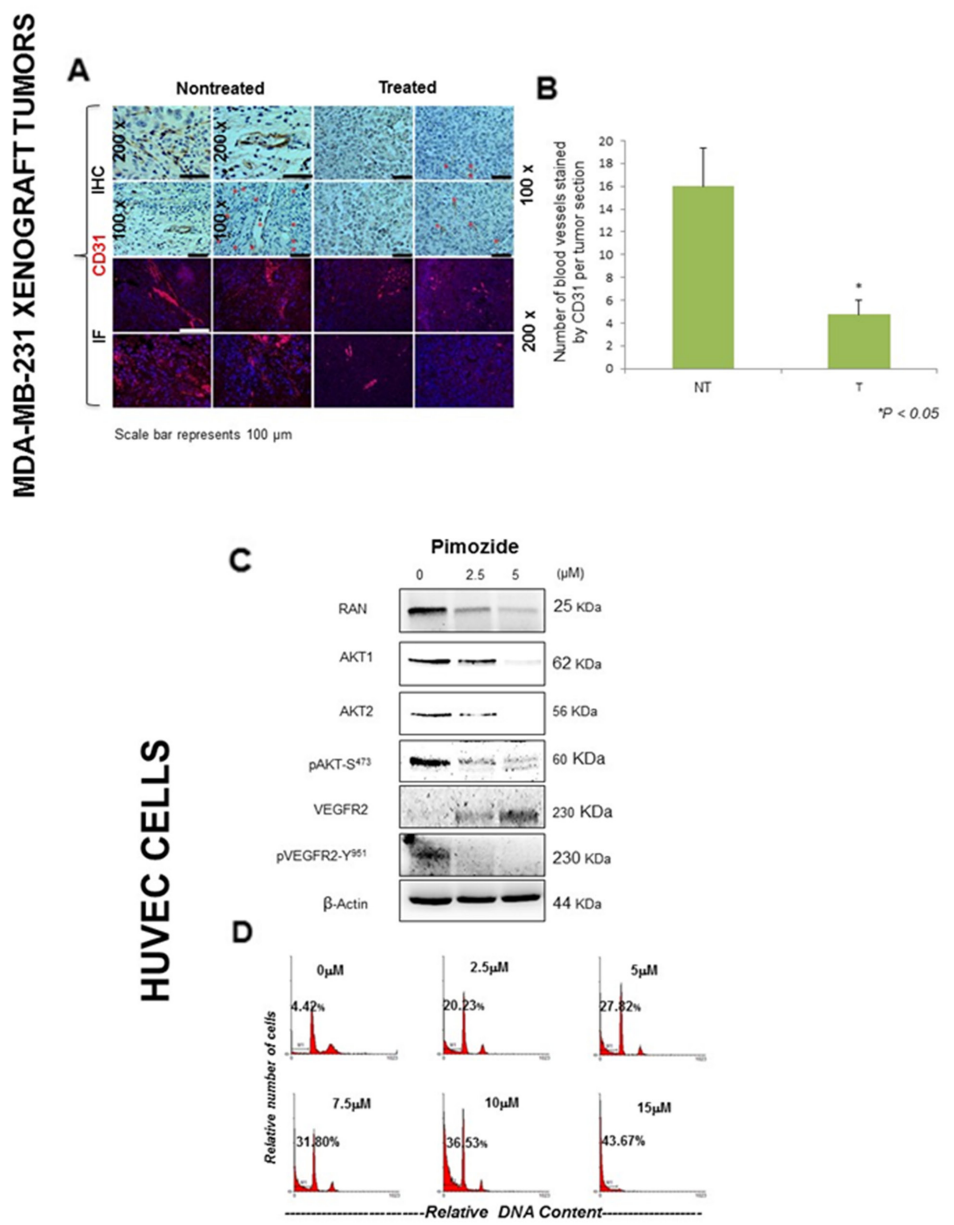
Pimozide inhibits the expression of angiogenesis related markers *in vivo* and induced apoptosis in HUVEC endothelial cells **(A)** Immunohistochemical and immunofluorescent detection of the endothelial cell marker CD31 in MDA-MB-231 tumors from untreated mice and mice treated with Pimozide (representative of 10 sections per group). Scale bar 100 μm. **(B)** Histogram showing numbers of blood vessels in untreated and Pimozide treated tumor sections, Pimozide-treated tumors exhibited a ∼three-fold reduction in CD31 staining, Student’s *t*-test ^*^P < 0.05 vs control. **(C)** Western blotting of AKT1, AKT2, pAKT, VEGFR2 and pVEGFR2 proteins in HUVEC cells treated with Pimozide for 24 hours. β-Actin was used as a loading control. Data shown are representative of three experiments performed. **(D)** DNA ploidy analysis by flow cytometry of HUVEC cells treated for 24 hours with Pimozide. Untreated control cells were run in parallel. Apoptosis was determined in flow cytometry by the percentage of hypodiploid (sub-G1) cells following cell cycle analysis. The percentage of cells with a DNA content less than G1 (sub-G1) is indicated in each histogram. The cell cycle profiles shown, with the sub-G1 population indicated, are representative of three experiments performed.

To confirm an anti-angiogenesis effect of Pimozide *in vivo*, the expression of proteins involved in angiogenesis was assessed in treated and untreated tumors, and *in vitro*, using MDA-MB-231 and HUVEC cells. Immunohistochemical and immunofluorescence staining of tumor sections from Pimozide-treated mice revealed dramatically reduced staining for CD31 (Figure 5A) and stained blood vessels by 78% (Student’s *t* test *P < 0.05*) (Figure 5B) compared to untreated mice. The relative RNA expression of *VEGFR1* and *VEGFR2* was dramatically reduced, by 4.33 fold and 1.66 fold respectively, in fibroblast cells after Pimozide treatment at 7.5 μM (Supplementary Figure 4B, available online). Likewise, the promoter activity of the *VEGFR2* gene in MDA-MB-231 and A549 cells was significantly reduced by Pimozide treatment between 5 and 20 μM after 24 hours, and was completely abolished with 20 μM Pimozide (Supplementary Figure 5, available online). Immunoblotting of HUVEC cells treated with Pimozide for 24 hours (Figure 5C), revealed that Ran, AKT1, and AKT2 protein expression had reduced by 73%, 97%, and 95% respectively, after treatment with 5 μM Pimozide (Student’s *t* test *P < 0.05*). Phosphorylation of AKT and VEGFR2 was also dramatically reduced, although VEGFR2 protein expression increased (Figure 5C). Moreover, Pimozide exerted anti-proliferative effects on HUVEC cells with an increased apoptosis index measured as the fraction of cells in the sub-G1 part of the cell cycle by flow cytometry after treatment for 24 hours (Figure 5D), and there was a significant (20%) increase in cell death with only 2.5 μM Pimozide.

### Pimozide prevents TGFβ-induced myofibroblast cell differentiation by increasing apoptosis and reducing αSMA formation

**Figure 6:**
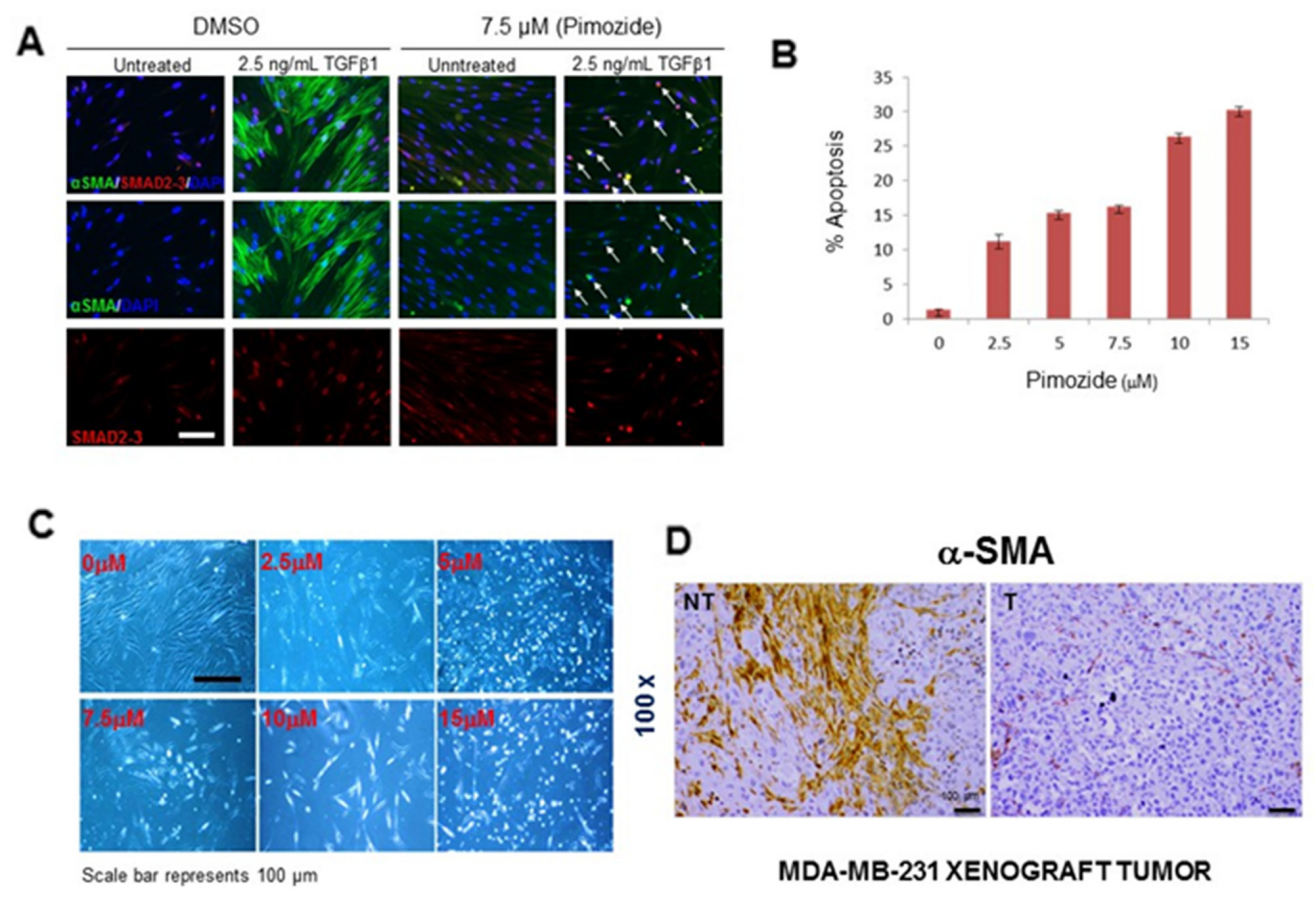
Pimozide suppresses fibroblast differentiation, reduces cell proliferation and increases apoptosis **(A)** Immunofluorescent detection of α-SMA/SMAD2-3 in untreated (DMSO) and treated fibroblasts with 7.5 μM Pimozide for 24 hours. The nuclei are stained blue (DAPI), smooth muscle in differentiated fibroblasts green, and SMAD2-3 red (nuclear staining). Primary human fibroblasts were pre-treated with 7.5 μM Pimozide, 1 hour prior to TGFβ1 treatment. Fibroblasts were stimulated to differentiate by addition of TGFβ1 for 72 hours. In vehicle control (DMSO)-treated cultures fibroblast differentiation was observed by the formation of polymerized smooth muscle actin filaments. However, in the presence of Pimozide, TGFβ1 was unable to promote the formation of these filaments. In Pimozide treated cells, SMAD2-3 is nuclear both in the control and TGFβ1-treated cultures. Exposure TGFβ1 treated fibroblasts to Pimozide resulted in increased apoptosis which is apparent from the increased number of condensed nuclei (white arrows). Scale bar 100 μm. **(B)** Cell cycle profile (Sub-G1) summarized using a histogram. Primary fibroblast cells were treated with different doses of Pimozide for 24 hours, and the percentage of cells in the sub-G1 phase of the cell cycle (dead and dying cells) was quantitated by flow cytometry. Data shown are means ± SD of three independent experiments, ^**^,*P < 0.01*, Student’s *t*-test. **(C)** Phase contrast micrograph showing the morphology of fibroblast treated with Pimozide at different doses for 48 hours. Scale bar 100 μm. **(D)** Immunohistochemical staining for α-SMA in MDA-MB-231 tumors from untreated mice (PBS) and treated mice (representative of 10 sections per group). Magnification 100x. Scale bar 100 μm.

Since myofibroblasts play an important role in metastasis by increasing the invasion and migration of cancer cells [27, 28], we further studied the effects of Pimozide treatment on myofibroblast cell differentiation, proliferation, and apoptosis. To address this process *in vitro*, we stimulated myofibroblasts with TGF-β1 (2.5 ng/mL). The expression of α-SMA protein increased after 24 hours and was highly elevated after 72 hours (Figure 6A), however treatment with Pimozide at 7.5 μM prevented fibroblast to myofibroblast differentiation by increasing apoptosis (Figure 6A, white arrow) as confirmed by cell cycle analysis (flow cytometry) with increasing apoptosis (Sub-G1) at increasing concentrations of Pimozide after 24 hours (Figure 6B). *In vitro* treatment of fibroblastic cells with increasing concentrations of Pimozide also increased apoptosis (Figure 6C). We also studied the effect of Pimozide on tumor associated myofibroblasts through immunohistochemical staining for αSMA in mouse tumors. This protein was completely depleted in breast tumors in mice treated with Pimozide compared to those in untreated mice (Figure 6D), and the treated mice also had fewer lung metastases (Supplementary Figure 2B, available online).

### High level of VEGFR2 and Ran tumor expression is correlated with shorter survival time of breast cancer patients

**Figure 7:**
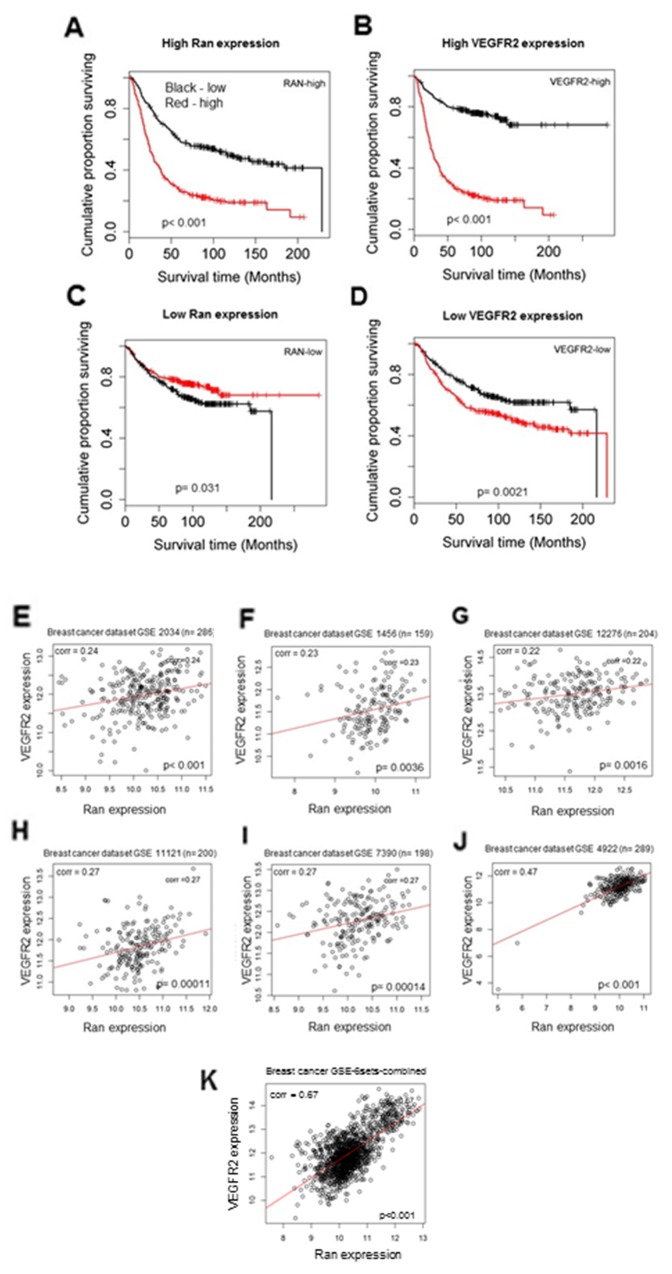
High level of Ran and VEGFR2 is correlated with shorter survival time of breast cancer patients **(A-D)** Kaplan-Meier plot of survival of breast cancer patients from five microarray datasets available in GEO database for *Ran* and *VEGFR2* mRNA. (A) Ran-high group stratified into high and low VEGFR2. The two curves were significantly different (Wald test χ^2^ = 67.03, 1 df, *P < 0.001* (P = 2.2e-16)). (B) VEGFR2-high group stratified into Ran high and low. The curves differed with a high degree of significance (χ^2^ = 138.61, 1 df, *P < 0.001* (P = 1e-16)). (C) Ran-low level group stratified into VEGFR2 high and low. The high group for VEGFR2 showed a significantly lower patient survival time (χ^2^ = 4.64, 1 df, P = 0.031). (D) VEGFR2-low group stratified into high and low Ran. The curves differed with a high degree of significance (χ^2^ = 9.5, 1 df, P = 0.0021). Only datasets that included at least 150 patients were included, and the correlations between relative mRNA levels of *VEGFR2* and *Ran* in each dataset were compared using Pearson’s correlation analysis (**E–J**). In all the datasets tested in this study GSE2034 (n = 286) (E), GSE1456 (n = 159) (F), GSE12276 (n = 204) (G), GSE11121 (n = 200) (H), GSE7390 (n = 198) (I), and GSE4922 (n = 289) (J), the relative mRNA levels of *VEGFR2* and *Ran* were significantly positively correlated (Pearson’s correlation; *P < 0.05*). (**K)**Association of mRNA levels of *VEGFR2* and *Ran* in GSE-6sets-combined breast cancer patients. The relative mRNA levels of *VEGFR2* and *Ran* were significantly positively correlated (Pearson’s correlation; *P < 0.05*).

Tumors were stratified according to mRNA expression of *Ran* and *VEGFR2*. A high level of expression for *Ran* and *VEGFR2* was associated with shorter patient survival time (Wald test, *P < 0.001*; Figure 7A, 7B), although this relationship was weaker in patients with low *Ran* and *VEGFR2* expression (Wald test, p= 0.031; Figure 7C, p= 0.0021; Figure 7D).

### Association between *VEGFR2* and *Ran* mRNA expression in human breast cancer

The mRNA levels for *Ran* and *VEGFR2* from six independent breast datasets were analyzed, revealing a statistically significant positive correlation between the mRNA levels of these two genes in all six individual datasets (Pearson’s correlation, *P < 0.001* (Figure 7, 7E-7J)). When the six datasets were combined (n = 1334) (Figure 7K), the positive association between *Ran* and *VEGFR2* mRNA levels was highly significant (Pearson’s correlation 0.67, *P < 0.001*). These results strongly demonstrate the positive association between *VEGFR2* and *Ran* expression in patient specimens.

## DISCUSSION

Several epidemiological studies have noted a lower rate of lung cancer among schizophrenic patients compared to the general population, even though these patients are more likely to smoke. Likewise, clinical data indicates that the use of medication for schizophrenia lowers the relative risk of cancer [5, 6, 10]. It has also been shown that the neuroleptic agents Pimozide and Thioridazine inhibit cancer growth in culture [17], either by targeting STAT3/STAT5 signaling pathways [18, 19] or by disrupting the Wnt/β-catenin signaling pathway [20] and more recently another group [29] reported that Pimozide kills lung, breast, and brain cancer cells *in vitro* [30], In this study we have attempted to elucidate further the mechanism underlying these clinical observations and identify a novel biological function for Pimozide.

This study is the first to report that an FDA-approved neuroleptic drug, Pimozide, which is already in clinical use, has multiple anticancer effects, since it inhibits cell proliferation, and has an anti-angiogenic and anti-metastatic activity. We found that Pimozide inhibited breast and lung cancer cell proliferation and sphere formation (data not shown) primarily by inducing apoptosis. These results are in agreement with other studies that showed an inhibitory effect of Pimozide on cell growth in breast cancer [17]. Pimozide also had a genotoxic effect on DNA by promoting DNA double-strand breaks measured by an increase of phosphorylation of histone H2AX (λ-H2AX), which is a marker of genomic instability [31], to a greater degree than the other FDA-approved chemotherapeutic drugs tested, Doxorubicin and Paclitaxel.

It was also demonstrated for the first time in this study that Pimozide acts as a potent anti-angiogenic modulator through the inhibition of the AKT and VEGF signaling pathways. Interestingly, the data showed that Pimozide affected the AKT signaling pathway by downregulation of phospho-AKT (Ser-473) and *AKT* gene expression. Previous studies have demonstrated that the effects of Pimozide on neurologic diseases are via antagonism of DRD2 [32], indeed, DRD2 is expressed in various cancer types [33, 34] and has been involved in regulating Wnt and AKT signaling [35] therefore, we postulate based on the data herein that the reduction of AKT signaling and *AKT* gene expression by Pimozide may result from inhibition of DRD2 receptor. This has been implicated in previous study which showed that downregulation of DRD2 whether through transient knockdown of the receptor or pharmacological antagonism can have cytotoxic effects towards cancer cells [36]. On the other hand, we have shown a potent anticancer effect of Pimozide, this may be attributed at least in part to Pimozide ability to engage other pathways that are not directly related to DRD2 antagonism. We observed decreased expression of *Ran* at mRNA and protein level *in vitro* and in an *in vivo* MDA-MB-231 xenograft model. It has been reported that typical and atypical antipsychotic drugs [37–40] can alter transcription particularly through altered binding of Fos and Jun family of transcription factors (TF) to their cognate AP-1 DNA binding site. We speculate that Ran expression may be directly regulated via these mechanisms or potentially via Pimozide driving expression of an unknown factor which can then decrease *Ran* mRNA expression and be responsible for decreased levels of Ran protein expression. However, further studies are needed to test these hypotheses.

The phosphorylation of VEGFR-2 plays an important role in promoting VEGF-induced tumor angiogenesis [41, 42], and we found that Pimozide also suppresses VEGF-induced HUVEC proliferation. It is well established that VEGF plays an important role in tumor angiogenesis by activating the proliferation and migration of endothelial cells during micro-vessel formation [43], and correspondingly we found that VEGF probably contributes to the growth of breast tumors both directly through its action on cancer cells and through its effect on angiogenesis as shown by a drastic reduction in blood vessel formation.

In cancer, endothelial cells play a pivotal role in regulating various aspects of vascular biology and pathology. Although VEGFR-1 and VEGFR-2 are structurally similar, they have distinct functions during angiogenesis. VEGFR-2 plays a vital role in activating the major downstream components responsible for cell growth, endothelial cell invasion, migration, differentiation, and embryonic angiogenesis [44, 45], whilst VEGFR-1 has no role in the proliferation and migration of endothelial cells [46]. Promoter analysis of the VEGFR-2 gene showed a clear dose-and time dependent inhibitory response to Pimozide (Supplementary Figure 5, available online), indicating that this and other neuroleptic agents could exert antitumor effects through disrupting the vasculature in addition to promoting apoptosis in the tumor cells themselves.

The production of MMPs by tumor cells promotes cellular migration and invasion through degradation of the extracellular matrix. The results of the present study show that Pimozide can downregulate MMPs-1 -2, and -14 (Figure 3C, 3E). MMPs are zinc dependent enzymes reported to be involved in the initial stages of invasion and metastasis of various tumor cells [47]. Activation of MMPs has been observed in a variety of metastatic tumor tissues, and hence numerous anticancer drug formulations focus primarily on attenuating the expression of these enzymes. Inhibition of MMP expression and activity is considered to be an early target for preventing cancer metastasis [48]. Our results demonstrated that an anticancer effect of Pimozide is associated with decreased MMP expression *in vitro* and *in vivo* (Figure 3C, 3E). This concurs with the finding that Ran silencing also downregulated MMP2 in MDA-MB-231 cells (Figure 3D). Hence Pimozide may prevent the migration of cancer cells at least in part through the down-regulation of MMP2 (Figure 3A, 3B).

We also found that Pimozide significantly inhibited MDA-MB-231 cell growth, migration, invasion and tumorigenicity *in vivo* and these effects were associated with suppression of EMT, a process involving the breakdown of cell-cell junctions and loss of epithelial polarity [49–51]. In our study, the expression of N-cadherin and Vimentin, and the transcription factors Twist, Slug, and Snail [52, 53] were reduced *in vitro*, and Vimentin expression was also reduced *in vivo* after Pimozide treatment (Figure 4D). Interestingly E-cadherin was unchanged during treatment suggesting that reversal of EMT was only partial or incomplete.

The present study also investigated the modulation of motility and expression of the FA protein, paxillin, and the cytoskeletal proteins myosin IIA and Arp3 after Pimozide treatment. These studies showed that Pimozide inhibits motility of breast and lung cancer cells *in vitro* and that there was a corresponding upregulation of these proteins and a change in the spatial relationship between FAs and actin filaments. There were also pronounced changes in the overall cytoskeletal architecture of these cells including a dramatic reduction in the number of cellular protrusions and an increase of actin stress fibers in response to Pimozide treatment suggesting a possible mechanism of action that depends on cytoskeletal distribution.

An anti-proliferative and anti-metastatic role for Pimozide is further supported by the finding that Pimozide suppresses the differentiation of fibroblasts to myofibroblasts. The latter are a component of the tumor microenvironment and help maintain tumor growth and invasion [54, 55]. This in turn points to an additional mechanism by which Pimozide can inhibit breast cancer tumor invasion. Myofibroblasts tend to promote tumor growth, especially in breast cancer, and hence could represent an additional therapeutic target.

**Figure 8:**
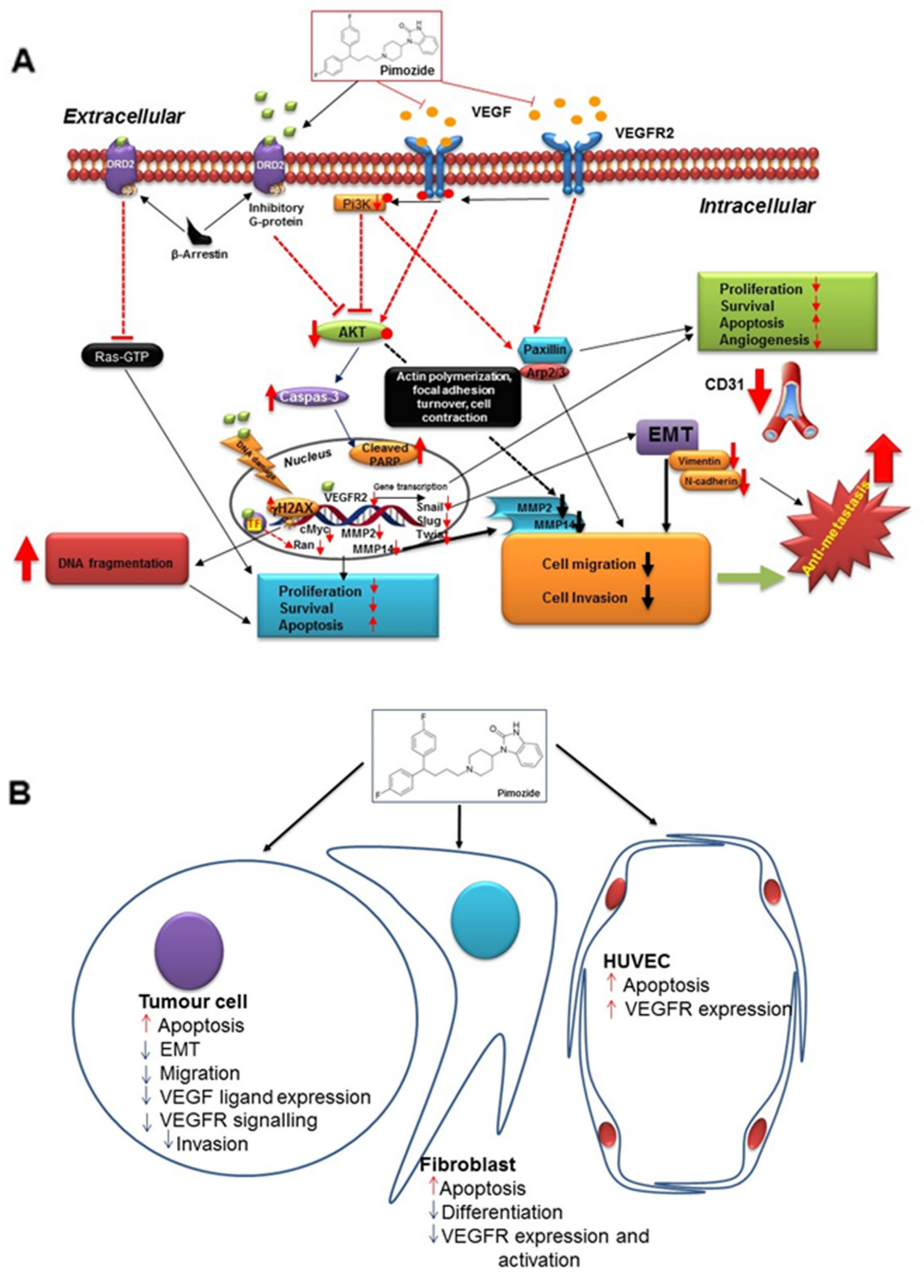
Summary figure of proposed pimozide mechanisms **(A)** Hypothetical representation of Pimozide signaling pathways involved in cell proliferation, survival, metastasis, and angiogenesis. **(B)** Schematic representation of Pimozide effects on tumor cells and the tumor microenvironment, including fibroblasts and HUVEC cells.

Collectively this study provides strong evidence for multiple molecular mechanisms (Figure 8A, 8B) through which Pimozide exerts anti-tumor activity in breast cancer cells and therefore could be a useful agent for treatment in the future.

## MATERIALS AND METHODS

### Cell culture

The MDA-MB-231 and MCF-7 breast cancer cell lines, A549 lung cancer cell line and viral packaging cell lines 293T (HEK) were obtained from the American Type Culture Collection (ATCC), Manassas, VA, US and maintained in culture medium (DMEM) containing 10% (v/v) fetal bovine serum (FBS) and 2 mM L-glutamine. MDA-MB-231-Luc (D3H2LN), was purchased from Cell Biolabs, INC (Cell Biolabs, San Diego, CA, USA). Human Umbilical Vein Endothelial Cells (HUVECs) were kindly donated by Dr. Andriana Margariti (Center of Experimental Medicine, QUB, Belfast, UK) and cultured with EGM-2 medium (Cambrex, Corporate, NJ, US). The Ras-transformed MCF10A derivative MCF10AT cell line (from Karmanos Cancer Centre, Detroit, MI, USA) was maintained in DMEM/F-12 medium containing 5% horse serum (v/v), 10 μg/ml insulin, 20 ng/ml epidermal growth factor (EGF), 100 ng/ml cholera toxin and 0.5 μg/ml hydrocortisone. Primary Human Foreskin Fibroblasts, purchased from Cascade Biologics - Thermo Fisher Scientific, Paisley, UK, were cultured in 1:1 (v/v) Dulbecco’s modified Eagle’s medium (DMEM)/Ham’s F-12 medium containing 10% (v/v) FBS and antibiotics (100 units of penicillin, 100 μg of streptomycin, 0.25 μg of amphotericin B, and 50 μg of gentamicin per ml). Human recombinant TGF-β1 (R&D, Minneapolis, MN) at 2.5 ng/ml with and without Pimozide at 7.5 μM, was used to treat human fibroblast on the next day. All cell lines were previously tested for mycoplasma contamination using MycoProbe Mycoplamsma Detection Kit (R&D systems, Abingdon, UK). Pimozide (Orap), Doxorubicin and Paclitaxel was purchased from Sigma Aldrich, dissolved and stored as indicated by the manufacturer’s, DMSO was purchased from Sigma Aldrich to dissolve all drugs and used as control.

### Transfection

Transfection was performed using GeneJuice^®^ (Promega, Southampton, UK) according to the manufacturer’s instructions. shScr, shRAN-1, shRAN-2 and shRAN-4, (Sigma-Aldrich, Dorset, UK) were used for Ran knockdown by Lentiviral infection as previously described [56].

### Luciferase reporter-gene assay

*In vitro* luciferase activity was performed as reported previously [57]. In brief, cells at 80 to 85% confluency were co-transfected with promoter *Renilla* luciferase plasmids with *Renilla* luciferase reporter (Promega, Hampshire, UK) for 24 hours. After lysis with Radio Immunoprecipitation Assay (RIPA) buffer, lysates were cleared by centrifugation for 15 minutes at 14000 rpm and the cell extracts were incubated with luciferase substrate for 30 minutes at room temperature, as indicated by the manufacture, then a 5 μl aliquot of each sample was quantified in a MicroLumat Plus LB96V luminometer. The ratio of luciferase fluorescence was normalized for the *Renilla* luciferase fluorescence to correct for variation within transfections. The results were the mean ± SD of the expression of three independent experiments.

### Real-Time polymerase chain reaction

Total RNA was isolated using the TRIZol reagent (Invitrogen) and reverse transcription was performed using SuperScript^™^ III first strand synthesis system (Invitrogen), according to the manufacturer’s instructions. The concentration of purified RNA was determined by the Agilent 2100 bioanalyzer using RNA 6000 NanoChips (Agilent, Waldbronn, Germany). The primers used for gene expression were purchased from R&D Systems (Supplementary Table 2, available online). As an internal standard, the RPLO (50S ribosomal protein L15) gene was chosen as a house-keeping gene. qRT-PCR was performed using the QuantiTect SYBR Green RT-PCR kit (Qiagen, Hilden, Germany) on a DNA Engine Opticon (Bio-Rad, Loanhead, UK). All reactions were performed with 500 ng of total RNA in a volume of 25 μl. Thermal cycling conditions were 30 minutes at 50°C, 15 minutes at 95°C, followed by 35 cycles of 30 seconds at 95°C, 30 seconds at 60°C, and 30 seconds at 72°C and finished with a melting curve. The expression level of each gene was quantified using the 2^-ΔΔCt^ method [58].

### Western blot analysis

Western blotting was performed as previously described [59]. Briefly, cells were lysed in RIPA buffer containing protease inhibitors. The samples were separated by SDS-PAGE and transferred onto a nitrocellulose membrane (Millipore, Watford, UK). The membranes were blocked with 5% (v/v) nonfat dried milk in PBS and subsequently incubated with primary antibodies anti-active caspas-3 (1:1000) from BD Pharmingen, anti-PARP (1:1000) from Cell Signaling, anti-Ran (1:2000), anti-MMP2 (1:100) from Millipore, anti-Snail (1:1000), anti-Vimentin (1:1000), anti-N-cadherin (1:1000), anti-E-cadherin (1:1000), anti-AKT1 (1:1000), anti-AKT2 (1:1000), anti-pAKT (1:2000), anti-VEGFR2 (1:1000) and anti-pVEGFR2 (1:1000) from Cell Signaling, overnight at 4° C. Bound antibodies were detected by horseradish peroxidases-conjugated secondary antibodies at 1:10000 at RT for 1 hour with enhanced chemiluminescence (Amersham Pharmacia Biotech, Chalfont, UK) for detection. Densitometry on scanned immunoblot images was performed using the ImageJ software.

### Apoptosis assays

Quantification of apoptotic cells was determined by flow cytometry as the percentage of cells in the sub-G1 region (hypodiploidy) of a cell cycle analysis as previously described [60]. Cell cycle profiles were generated using manually drawn gates with Cyflogic software.

### MTT cell proliferation and survival assay

Cells were seeded at a cell density of 3000 cells/well in a 96-well plate in triplicates and allowed to grow for 24 hours and before Pimozide at different doses was added. The MTT (3-(4,5-dimethylthiazol-2-yl)-2,5-diphenyltetrazolium bromide) dye uptake method was used as previously described [61] to assess cell survival 24, 48 and 72 hours post-treatment.

### Boyden chamber migration and invasion assays

Migration and invasion assays were performed as previously described [62]. Briefly, 5000 and 50000 cells in serum-free media were seeded into the upper chamber (Millipore) on top of the membrane with or without a Matrigel coating, respectively, for migration and invasion assays. The cells were allowed to migrate or invade towards the bottom layer with 10 ng/ml hepatocyte growth factor (HGF) as chemoattractant for 24 hours. Cells in the bottom of the membrane were fixed and stained with crystal violet.

### Cell adhesion assay

For the cell adhesion assay, 4000 cells suspended in serum-free media with or without 10 ng/ml HGF were seeded per well in a 96-well plate coated with fibronectin and the cells were allowed to settle for 30 minutes. Non-adherent cells were then removed by washing 4 times with PBS. Adherent cells were fixed and stained with crystal violet. The excess dye was washed away, and the retained dye was extracted. The absorbance at 595nm was measured in a microplate reader.

### Xenograft mouse models

Animal procedures in this study complied with UK Animal Use in Research Guideline (UK Directive) and the European Union (European Directive 2010/63/EU) guidelines on animal Experimentation for the Protection and Humane use of Laboratory Animals, and were conducted at an accredited Animal Experimentation Facility (MBC, Queen’s University, UK). Procedures were approved by the Ethics Committee at Queen’s University. Female C57/BL6 nude mice (8-weeks old), were kept and handled according to Institutional Guidelines, complying with UK legislation under 12/12 hours light/dark cycle at a temperature of 22°C, and received a standard diet and acidified water ad libitum. MDA-MB-231-Luc cells (2 × 10^6^) were injected subcutaneously in 100 μl phosphate-buffered saline together with 100 μl Matrigel (Becton Dickinson) into one mammary fat pad of each mouse. Mice were randomly assigned to cohorts of ten mice each, and were grouped in four groups, Group-1 (G1) without cell implantation and without treatment; Group-2 mice received cells and were not treated with Pimozide (G2NT); Group-3 mice were implanted with cells and treated a day earlier prior to implantation (G3TE); Group-4 mice were injected with cells and treated late when tumors were palpable (G4TL), G3TE & G4TL mice received an i.p. injection of 20 mg/kg Pimozide or the same volume of vehicle (PBS) as the G2NT animals. The shortest and longest diameter of the tumor were measured with calipers at the indicated time intervals, and tumor volume (mm^3^) was calculated using the following standard formula: (the shortest diameter)^2^ × (the longest diameter) × 0.5. Animal body weight and any sign of morbidity were monitored. Drug treatment lasted 4 weeks. Animals were killed 24 hours after the last drug administration according Schedule 1 killing procedures, and then tumors were carefully removed, weighed, and analyzed. A total necropsy involving tumors and distinct organs was carried out.

### Histochemical analysis

Tumor tissue samples were fixed in 4% (w/v) buffered paraformaldehyde and embedded in paraffin. Wax tissue sections (4-5 μm) were deparaffinized and hydrated in graded ethanol and distilled water. Endogenous peroxidase activity was blocked using methanol and 3% H_2_O_2_ for 30 minutes as previously described [59]. Histological sections were counterstained with hematoxylin and eosin (H&E), others were then incubated at 4°C with different antibodies: rabbit anti-human Ki67 monoclonal antibody (clone SP6) (1:1000 dilution, Abcam), anti-active human cleaved-caspase-3 (1:200 dilution for IHC & 1:100 for IF, Cell Signaling) rabbit polyclonal antibody that specifically recognized the active ∼20-kDa subunit (Cell Signaling), anti-human Ran (1:1000, Millipore), anti-human MMP2 (1:100 dilution, Millipore), anti-AKT (Pan) (1:300, Cell Signaling), anti-AKT1, anti-AKT2, anti-phospho-AKT (1:1000, Cell Signaling), anti-human CD31 (1:50 for IHC and 1:20 for IF, Abcam) and anti-α-smooth muscle actin (α-SMA) (1:50 for IHC and 1:100 for IF, Abcam). After washing with PBS, sections were incubated with biotinylated anti-rabbit IgG antibody (BD Pharmingen) for 60 minutes at room temperature and washed with PBS. Then, sections were incubated with streptavidin horseradish-peroxidase (Vector Laboratories) for 60 minutes in a moist chamber, washed with PBS, and sites of peroxidase activity were visualized using 3,3′-diaminobenzidine tetrahydrochloride solution (DAB). Sections were subsequently counterstained with hematoxylin Mayer’s & Eosin (Sigma). Staining was analyzed with a Nikon Eclipse 400^®^ microscope and Metamorph^®^ software (Molecular Devices Corporation).

### Immunofluorescence staining

Human breast and lung cancer cell lines MDA-MB-231, MCF7 and A549 were seeded onto fibronectin-coated (2.5 μg/cm^2^) glass coverslips (15000 cells/coverslip) in a 24-well plate. Following 48 hours incubation, cells were stained in order to analyse cytoskeletal organization as previously described [24], they were first washed once in cytoskeletal buffer (CB: 150 mM NaCl, 5 mM MgCl2, 5 mM EGTA, 5 mM glucose, 10 mM 2-(N-morpholino) ethane-sulfonic acid, (pH 6.1) prior to fixation at 37°C for 20 minutes in CB buffer with 3.7% (w/v) paraformaldehyde. Cells were then further washed in CB buffer before being permeabilized with 5% (v/v) Triton-X100 in CB buffer for 2 minutes and blocked with 5% (v/v) goat serum in CB buffer for 60 minutes. Samples were incubated with primary antibodies against either myosin IIA (Covance), paxillin (Abcam) or Arp3 (Abcam) at a dilution of 1:1000, 1:100 and 1:200, respectively in blocking solution, 5% (v/v) goat serum in CB buffer for 45 minutes at room temperature. After washing three times with blocking solution, cells were incubated with their corresponding secondary antibodies (anti-rabbit and anti-mouse antibodies labelled with FITC) in blocking solution for 45 minutes at room temperature. For actin staining, rhodamine phalloidin was also added with secondary antibodies at a concentration of 0.6 μM. Staining for γH2AX, αSMA, SMAD2/3 and TGFβ1, was carried out on MDA-MB-231 cells grown in glass coverslips with and without treatment. Cells were incubated with primary antibodies anti-γ-H2A.X (phospho S139) antibody [9F3] (1:1000, Abcam), anti-αSMA (1:100, Abcam), anti-Smad2 and anti-Smad3 (1:1000, Abcam) and anti-TGFβ1 antibody (1:100, Abcam), respectively. Cells were stained with DAPI to visualise the nucleus. After washing three times with blocking solution, cells were incubated with their corresponding secondary antibodies (anti-rabbit and anti-mouse antibodies labelled with FITC). After washing with blocking solution, coverslips were rinsed with water and mounted in vectashield antifade mounting medium (Vector Laboratories), and viewed using a confocal microscope (TCS SP5 II Confocal, Leica) using a HCX PL APO 63x/1.4–0.6 oilCS objective at 20% argon laser intensity.

### Motility wound healing assay

The wound closure assay was performed to determine changes in cellular motility. For this assay, either 1×10^5^ MCF7 or 6×10^4^ MDA-MB-231 cells were seeded into each well of a 24-well tissue culture plate and left to grow for 48 hours to reach 90-95% confluency. Confluent monolayers were scratched with a sterile pipette tip prior to addition of 7.5 μM Pimozide in normal medium for the treated cells. Wound closure was monitored by collecting digitalized images using the Cell-IQ automated image capture system (Chip-Man Technologies) on preselected fields using phase contrast microscopy. Analysis of the collected data was performed using the Cell-IQ Analyser Software, to generate percentage of wound closure over time. Quantification of the closed area after 24 hours was used to generate data for this assay and normalized as a percentage of the closed area in the untreated control.

### Connectivity mapping analysis

Gene expression connectivity mapping is an advanced bioinformatics technique to establish the connections among genes, drugs, and diseases via gene expression profile similarities. The statistically significant connections map (sscMap) framework [63–65] was used to search for small molecule compounds with potential to suppress the expression of *Ran*. Briefly, the reference gene expression profiles included in the sscMap core database were based on Affymetrix HG-U133A microarray platform, and Affymetrix Probset IDs were used as gene identifiers in those profiles. To make connections between *Ran* expression and candidate small molecules, gene symbols including *Ran* were mapped to the Affymetrix Probset IDs using the corresponding Affymetrix annotation file. The connections between *Ran* expression and the reference profiles of each compound were assessed with the scoring and statistical testing procedures, as previously described [64].

### Statistical analysis

Data are presented as the mean ± standard deviation (SD) or ± standard error (SE). The Student’s *t*-test and one-way ANOVA were used to assess the significance of independent experiments. Survival analysis was performed using Kaplan-Meier plots and significance of differences were tested using the Wald test. A p-value < 0.05 was considered significant in all statistical analyses. Differences in expression levels between groups/samples in human specimens were analyzed using the χ^2^, Fisher exact, or Mann-Whitney U tests, and where applicable, Pearson’s correlation, using SPSS 19.0 software (IBM, Armonk, NY).

### Analysis of breast cancer microarray data

Five breast cancer datasets [GSE1456 [66], GSE2034 [67], GSE4922 [68], GSE7390 [69], GSE12276 [70]], obtained from Gene Expression Omnibus (GEO) (https://www.ncbi.nlm.nih.gov/geo/), each consisting of more than 150 patients with their corresponding microarray gene expression data and survival data available in GEO were included in this study. The datasets were pre-processed as previously described using R and Bioconductor for normalization [71]. Information about the patients and breast cancer samples can be found on the GEO website. All these data sets were used to generate the Kaplan-Meier curves. The combined breast cancer dataset consisted of 1096 patients. The patients were first split into two groups by the median expression value of RAN (or VEGFR2), resulting in a RAN-high group and a RAN-low group. Then within each group the patients were split by the median expression value of the other gene, VEGFR2 (or correspondingly RAN), into two sub-groups, for which two Kaplan-Meier curves were created. Wald test were used to assess if they were significantly different. The low or high expression status is determined by the median expression level, above which is considered as “high” and below the median is “low” expression. The correlation in expression between Ran and VEGF2 was assessed using Pearson’s correlation analysis, based on the same sets of GEO data series used to generate Kaplan-Meier curves with an additional data series GSE11121 [72]. The latter was not used in the combined survival analysis, because GSE11121 has metastasis-free survival data rather than relapse-free survival data as the other 5 data series.

## SUPPLEMENTARY MATERIALS FIGURES AND TABLES


