# Supplementary material for: The anti-psychotic drug pimozide is a novel chemotherapeutic for breast cancer
Source: Oncotarget. 2018 Oct 9;9(79):34889–910. doi: 10.18632/oncotarget.26175 (PMC6201850; doi:10.18632/oncotarget.26175)
Supplement: Supplementary file 1 [file oncotarget-09-34889-s001.pdf]

The anti-psychotic drug pimozide is a novel chemotherapeutic for breast cancer

SUPPLEMENTARY MATERIALS

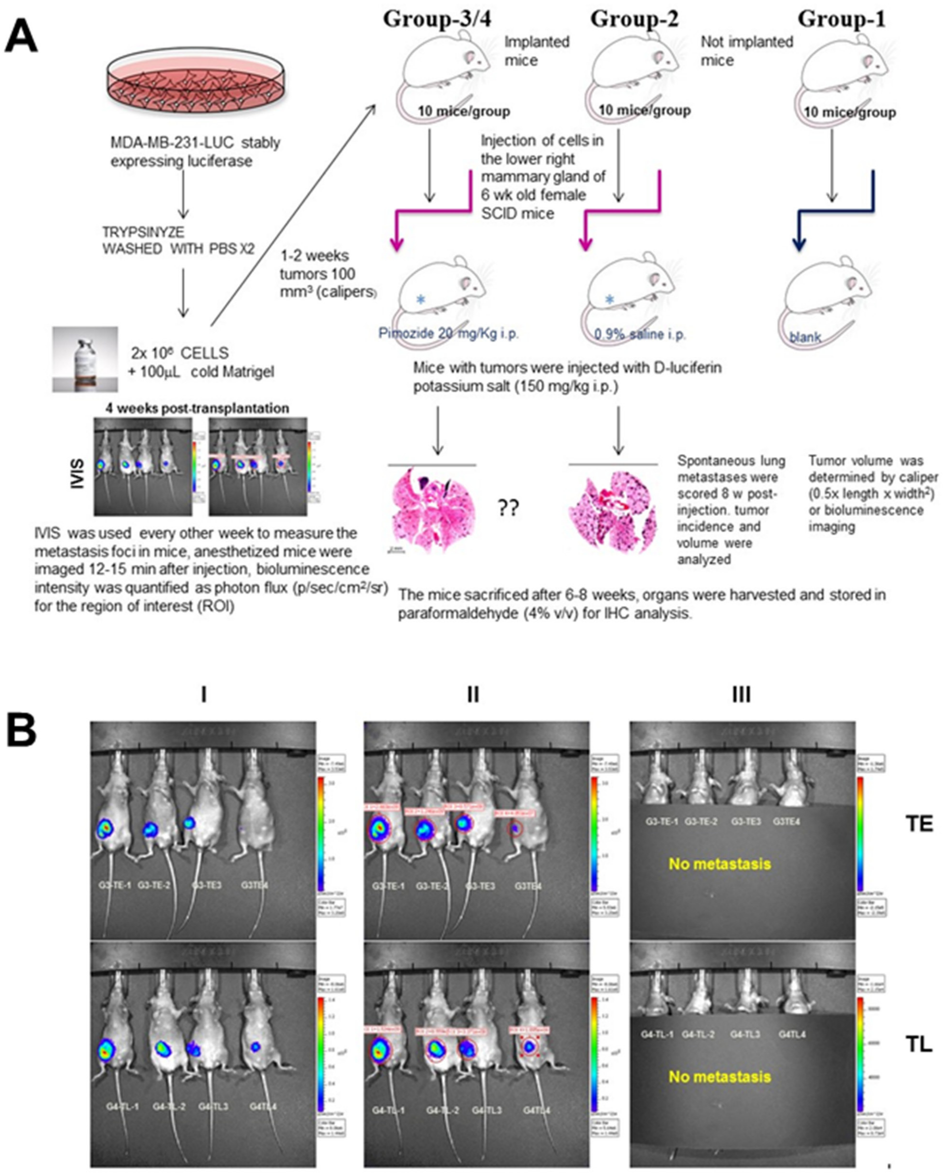

Supplementary Figure 1: (A) MDA-MB-231 breast cancer xenograft mouse model and study-design protocol. MDA-MB-231-Luc (D3H2LN) were grown *in vitro*, mice were grouped as Group-1, Group-2, Group-3 and Group-4 (a cohort of 10 mice), breast cancer cells expressing luciferase were implanted in one mammary fat pad of Group-2, Group-3 and Group-4, Group-1 used as control. Group-2 were treated with phosphate buffered saline (PBS), however Group-3 and Group-4 were treated with 20 mg/kg of Pimozide, early treatment for Group-3 and late treatment for Group-4. Mice were sacrificed, and lungs were removed and fixed in paraformaldehyde 4% (w/v), posterior tissues were stained with hematoxylin and eosin (H&E) for metastases detection in lung or immunostaining in tumors. **(B)** *In vivo* bioluminescence imaging system (IVIS) of MDA-MB-231-Luc (D3H2LN) xenografts in SCID mice. **I-** Ventral images taken over time from representative mice (4 out of 10 mice imaged) of G3TE and G4TL. **II-** Images shows the Region of Interest (ROI) in localized tumors from representative mice (4 out of 10 mice imaged). Pseudo color scale bars were consistent for all imaged ventral views to show relative changes at metastatic sites over time. **III-** IVIS system did not detect metastatic foci in G3TE and G4TL.

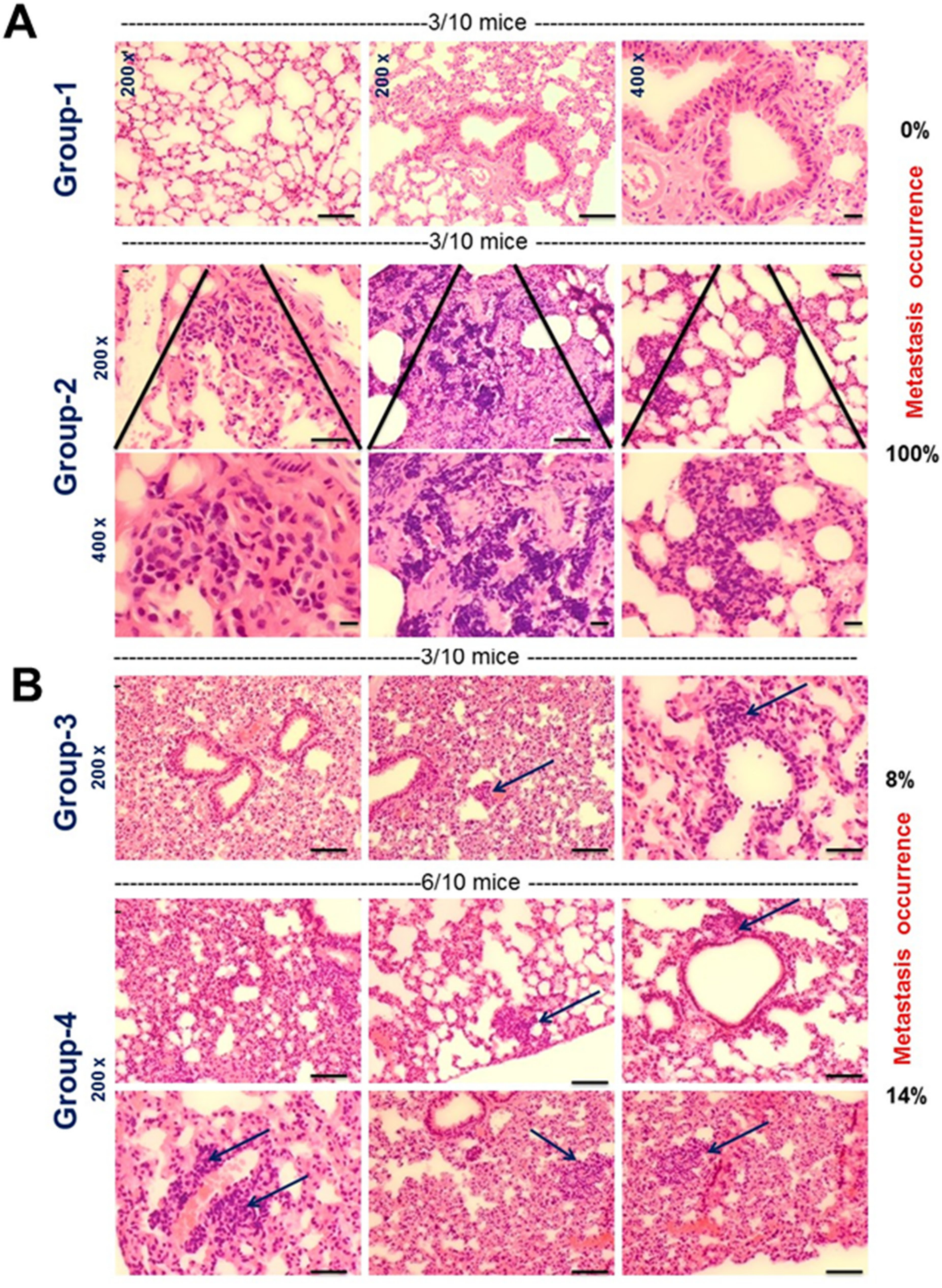

Supplementary Figure 2: Pimozide reduces the number of lung metastasis in a MDA-MB-231 mice xenograft model system. Lung metastasis occurrence in MDA-MB-231 xenograft model. Mice (Group-1, Group-2, Group-3 and Group-4) were sacrificed and 3 out of 10 representative mice lung (Group-1, Group-2 and Group-3) and 6/10 (Group-4), were fixed in paraformaldehyde 4% (w/v) and stained with hematoxylin & eosin (H&E). The occurrence of metastases was much higher in nontreated mice 100% in (Group-2) compared to treated mice 8% in (Group-3) and 14% in (Group-4). Group-1 (blank mice) were free of metastasis.

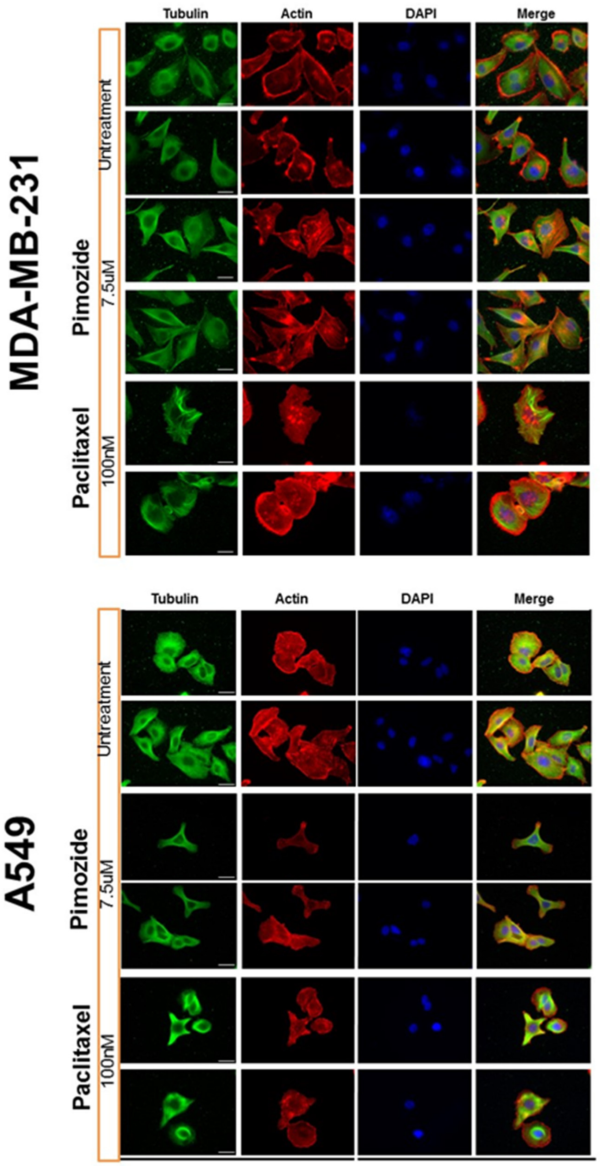

Supplementary Figure 3: Pimozide and Paclitaxel differentially affect microtubule organisation or nuclear condensation in MDA-MB-231 and A549 cancer cells. Breast cancer MDA-MB-231 cells treated either with 7.5 µM Pimozide, 100 nM Paclitaxel or control (DMSO) were grown for 48 hours on fibronectin-coated coverslips prior to washing, fixing and immunostaining for tubulin together with actin and FITC secondary labelled antibodies or for actin with rhodamine-phalloidin, before mounting and viewing using confocal microscope (TCS SP5 II Confocal, Leica) by using HCX PL APO 63x/1.4–0.6 oil CS objective at 20% argon laser intensity. Scale bar 100 µm. Paclitaxel resulted in significant disorganisation of the cells microtubular and actin cytoskeleton and caused extensive bundling throughout the cytoplasm in MDA-MB-231 and A549 cell lines. Significant increases in nuclear condensation could also be seen in both cell lines. Pimozide caused minor defects in the microtubule organisation or nuclear condensation with less than 10% of the cells affected in both MDA-MB-231 and A549 cell lines. Data shown are representative of three experiments performed.

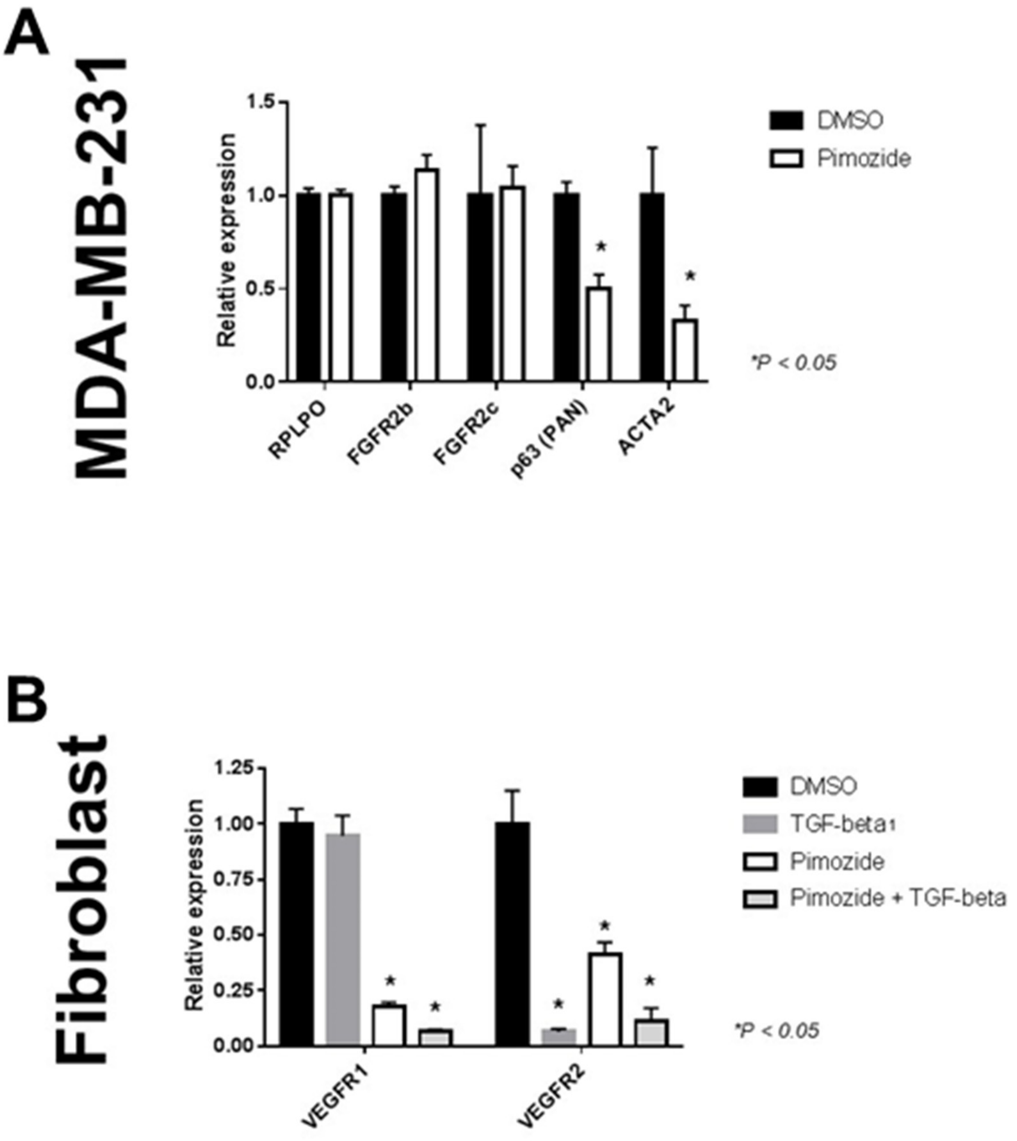

Supplementary Figure 4: (A) Pimozide reduced the RNA expression of other EMT markers *p63*, and *ACTA2* and not *FGFR2b*, *FGFR2c*. Relative mRNA of *p63*, *FGFR2b*, *FGFR2c* and *ACTA2* in MDA-MB-231 cells either untreated or treated with Pimozide at 7.5 µM for 24 hours. Data shown are representative of three experiments performed. (Student's *t* test $^{*}P < 0.05$). **(B)** Pimozide dramatically reduced the RNA expression of *VEGFR1* and *VEGFR2* in fibroblasts. Relative RNA expression of *VEGFR1*, *VEGFR2* in Fibroblast cells either untreated (DMSO) or treated with Pimozide at 7.5 µM for 24 hours. Pimozide reduced expression of both *VEGFR1* and *VEGFR2*, however only *VEGFR2* mRNA was strongly reduced in presence of TGFβ1 alone and had no effect on *VEGFR1* expression in Fibroblasts. Data shown are representative of three experiments performed. (Student's *t* test $^{*}P < 0.05$).

Supplementary Table 1: Summary of apoptosis (sub-G1) data

[Pimozide] μM	MDA-MB-231	A549
0	0.93	0.97
7.5	12.54	16.30
10	16.71	19.69

MDA-MB-231 and A549 cells were treated with different doses of Pimozide for 24 hours, percentage of apoptosis is presented.

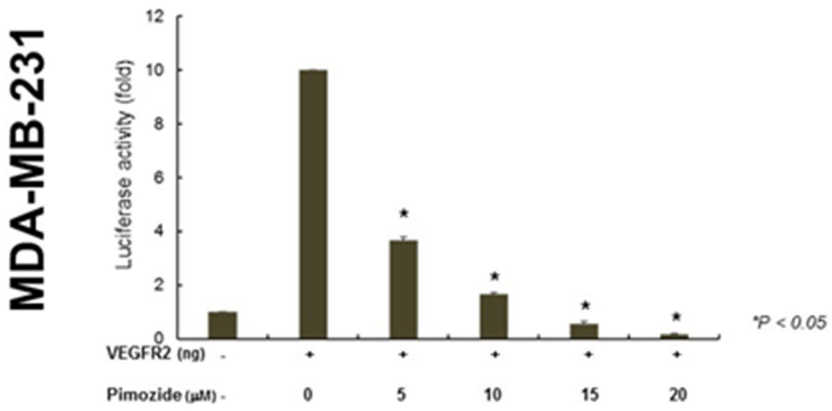

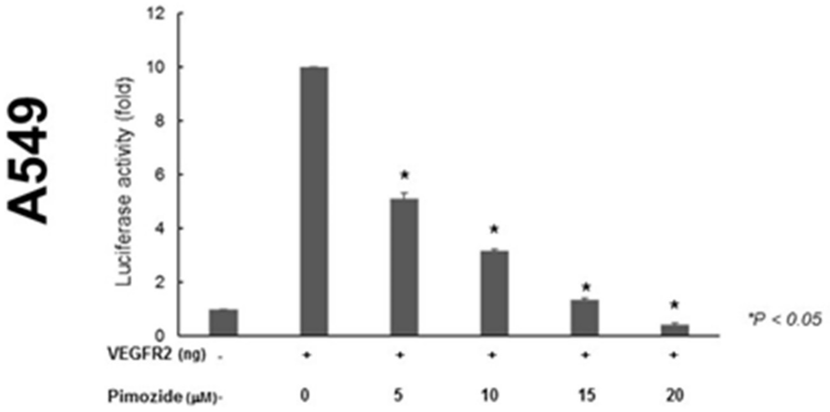

Supplementary Figure 5: Inhibition of the VEGFR-2-dependent transcriptional activity by Ran. MDA-MB-231 and A549 cells were co-transfected with 500 ng of VEGFR-2-Luc vector, 500 ng of a VEGFR-2 inserted expression vector (pGL3/VEGFR-2) and increasing concentrations of Pimozide (0, 5, 10, 15 and 20 μM). The results shown are representative of at least three independent experiments. The values are expressed as the means ± SD (Student's t test $^{*}P < 0.05$).

Supplementary Table 2: Primer sequences for RT-PCR and qPCR used in the study

Gene	Forward primer 5′---------->3′	Reverse primer 5′---------->3′	PCR-type
AKT1	CTGTCATCGAACGCACCTT	GTCTGGATGGCGGTTGTC	qPCR
AKT2	GAGGTCATGGAGCACAGGTT	CTGGCCGAGTAGGAGAACTG	qPCR
AKT3	GGATCACAGATGCAGCTACC	GTAGAAAGGCAACCTTCCACAC	qPCR
cMyc	CACATCAGCACAACTACGCAGCGC	CTCAGGACTCTGACACTGTCCAAC	qPCR
Snail	CCTCCCTGTCAGATGAGGAC	GTTCCTTATGGAGTCGGACC	qPCR
Twist	GGAGTCCGCAGTCTTACGAG	GGAGATGGTCCAGGAGGTCT	qPCR
cMet	CATGCCGACAAGTGCAGTA	TCTTGCCATCATTGTCCAAC	qPCR
Ran	AAAACGACCTTCGTGAAACG	TCAGTCCACCGAATTTCTCC	qPCR
N-cadherin	GCGTCTGTAGAGGCTTCTGG	GCCACTTGCCACTTTTCCTG	qPCR
N-cadherin	AGGGTGGACGTCATTGTAGC	CTGTTGGGGTCTGTCAGGAT	RT-PCR
Vimentin	TGTCCAAATCGATGTGGATGTTTC	TTGTACCATTCTTCTGCCTCCTG	qPCR
Vimentin	TCTCTGAGGCTGCCAACCG	CGAAGGTGACGAGCCATTTCC	RT-PCR
Zo-1	CGGTCCTCTGAGCCTGTAAG	GGATCTACATGCGACGACAA	RT-PCR
β-Actin	AGAGCTACGAGCTGCCTGAC	AGCACTGTGTTGGCGTACAG	RT-PCR
MMP1	TTACATCGT TTGCGGCTCATGAA	CGGACTTCATCTCTGTCGGCTAAT	qPCR
MMP14	TACTTCCCAGGCCCCAAC	GCCACCAGGAAGATGTCATT	qPCR
VEGFR1	CGGAAGGAAGACAGCTCATC	CTTCACGCGACAGGTGTAGA	qPCR
VEGFR2	GGCGGTGGTGACAGTATCTT	TCTCCGGCAAGCTCAAT	qPCR
RPLPO	ATCAACGGGTACAAACGAGTC	CAGATGGATCAGCCAAGAAGG	qPCR
FGFR2b	CCTGCCAAAACAGCAAGC	AAGACCCCTATGCAGTAAATGG	qPCR
FGFR2C	ACACCACGGACAAAGAGATT	GGCGATTAAGAAGACCCCTA	qPCR
ACTA2	CTGTTCCAGCCATCCTTCAT	TCATGATGCTGTTGTAGGTGGT	qPCR
P63(PAN)	GACAGGAAGGCGGATGAAGATAG	TGTTTCTGAAGTAAGTGCTGGTGC	qPCR
